# Heteroepitaxial passivation of Cs_2_AgBiBr_6_ wafers with suppressed ionic migration for X-ray imaging

**DOI:** 10.1038/s41467-019-09968-3

**Published:** 2019-04-30

**Authors:** Bo Yang, Weicheng Pan, Haodi Wu, Guangda Niu, Jun-Hui Yuan, Kan-Hao Xue, Lixiao Yin, Xinyuan Du, Xiang-Shui Miao, Xiaoquan Yang, Qingguo Xie, Jiang Tang

**Affiliations:** 1 Wuhan National Laboratory for Optoelectronics (WNLO), Huazhong University of Science and Technology (HUST), 430074 Wuhan, China; 2 School of Optical and Electronic Information, Huazhong University of Science and Technology (HUST), 430074 Wuhan, China; 3 College of Life Science and Technology, Huazhong University of Science and Technology (HUST), 430074 Wuhan, China

**Keywords:** Materials science, Materials for devices, Electronic devices, Optics and photonics

## Abstract

X-ray detectors are broadly utilized in medical imaging and product inspection. Halide perovskites recently demonstrate excellent performance for direct X-ray detection. However, ionic migration causes large noise and baseline drift, limiting the detection and imaging performance. Here we largely eliminate the ionic migration in cesium silver bismuth bromide (Cs_2_AgBiBr_6_) polycrystalline wafers by introducing bismuth oxybromide (BiOBr) as heteroepitaxial passivation layers. Good lattice match between BiOBr and Cs_2_AgBiBr_6_ enables complete defect passivation and suppressed ionic migration. The detector hence achieves outstanding balanced performance with a signal drifting one order of magnitude lower than all previous studies, low noise (1/*f* noise free), a high sensitivity of 250 µC Gy _air_^−1^ cm^–2^, and a spatial resolution of 4.9 lp mm^−1^. The wafer area could be easily scaled up by the isostatic-pressing method, together with the heteroepitaxial passivation, strengthens the competitiveness of Cs_2_AgBiBr_6_-based X-ray detectors as next-generation X-ray imaging flat panels.

## Abstract (web summary)

Ionic migration degrades not only the characteristics of halide perovskite solar cells, but also those of perovskite X-ray detectors. Here Yang et al. employ heteroepitaxial BiOBr to passivate Cs_2_AgBiBr_6_ double perovskite, which suppresses ionic migration and obtain high performance X-ray detectors.

## Introduction

X-ray detection is significant for medical imaging, security inspection and nondestructive examination of industrial goods etc^1^. Recently, metal halide perovskites have demonstrated excellent X-ray detection performance, due to the high X-ray attenuation coefficient, defect tolerance nature, and large mobility-lifetime product (*μτ*)^2–5^. Huang and co-workers pioneered the use of MAPbBr_3_ single crystals for X-ray detection and achieved an impressive sensitivity of 21000 μC Gy_air_^−1^ cm^−2^ toward 8 keV X-rays^3,4^. Park and co-workers reported the flat-panel detectors utilizing printable CH_3_NH_3_PbI_3_ films, obtaining a spatial resolution of 3.1 line pair per millimeter (lp mm^−1^)^6^. In parallel, in order to address the lead toxicity issue, we advocated Cs_2_AgBiBr_6_ as a promising non-toxic alternative with similar key features as lead halide perovskites, including a large *μτ* product of about 10^–3^ cm^2^ V^−1^, high X-ray absorption coefficient and larger resistivity (10^9^ to 10^11^ Ω cm)^7^. Cs_2_AgBiBr_6_ single crystal-based detector achieved a moderate sensitivity of 105 µC Gy_air_^−1^ cm^–2^ and a low detection limit of 59.7 nGy_air_ s^−1^, which is comparable to the best-performing lead halide perovskite X-ray detectors^5^. Cs_2_AgBiBr_6_ thus hold great promise toward direct X-ray detection applications.

Area scalability and ionic migrations are current two bottlenecks of perovskite X-ray detectors for their further employment in X-ray imaging. Generally, considering the X-ray refractive index as about 1.0 and difficulty to focus, large-area X-ray detectors with the size comparable to the object are necessary for imaging applications. Polycrystalline films capable of area-scalable fabrication are thus a feasible route toward such applications.

Equally important is the suppression of notorious fast ionic migration in metal halide perovskites. Notably, stable signal output, low noise, and high spatial resolution are principally regarded as the indispensable figures of merits for the X-ray imaging system. Both signal output and noise are directly related to the ionic migrations within perovskites. The ionic migrations render the baseline drifting, influence the signal recording and trigger overflow error^5^. They can also cause ionic conductivity, increase dark current and enlarge the shot noise^8^. Within polycrystalline films, the ionic migrations are even more severe. Although small bias (lower than 50 V) or even zero bias has been adopted to alleviate ionic migrations, the signal baseline of perovskite-based detectors is still drifting^3–6^. The use of small bias also concomitantly sacrifices the charge collection efficiency, increases signal crosstalk between pixels and negatively influences the spatial resolution^3,6^. It is thus significant but highly challenging to produce ionic migration-free polycrystalline thick films for X-ray imaging.

The established knowledge about the nature of ionic migration is the drift of halogen ions from lattice points to halogen vacancies, and ionic migration mostly occurs along the grain boundaries through the defective sites^9^. Thereby, passivating the grain boundary is the key to eliminate ionic migrations. Several strategies have been raised to passivate the grain boundary, such as employing PCBM, K^+^ or pyridine molecules, but still cannot suppress ionic migrations to a level as in single crystal counterparts^9–11^. We take the view that heteroepitaxial growth of wide band gap semiconductors could suppress dangling bonds and passivate the grain boundaries of perovskites^12^, as demonstrated in III-V and II-VI semiconductors (GaN/AlN, CdSe/CdS etc.)^13,14^. Beyond that, heteroepitaxial layer can further serve as solid barriers to block ionic migration. Epitaxial growth of perovskite onto substrates like mica, SrTiO_3_, NaCl has indeed demonstrated excellent optoelectronic properties, but the other interfaces without heteroepitaxy are still troubled by surface defects^15–17^. As far as we are concerned, the reports about the heteroepitaxial growth of other semiconductors onto metal halide perovskites for passivation are rare.

Here we demonstrate the fabrication of Cs_2_AgBiBr_6_ wafer, suppression of ionic migration via in situ heteroepitaxial growth of BiOBr, and sensitive and stable X-ray imaging applications. An isostatic-pressing method was used to synthesize Cs_2_AgBiBr_6_ wafers with upscaling capability, satisfying the requirements for large-area imaging applications. Thanks to the good lattice match between BiOBr and Cs_2_AgBiBr_6_ as well as convenient in situ formation of BiOBr, the epitaxial BiOBr serves as passivation layers and physical barriers to suppress the defects at grain boundaries and eliminate ionic migrations, as evidenced by that the measured activation energy of ionic migration for Cs_2_AgBiBr_6_ polycrystalline wafer is even slightly higher than Cs_2_AgBiBr_6_ single crystals and other lead-based perovskites. The about 20 cm^2^ Cs_2_AgBiBr_6_ wafer-based detector exhibits low noise (1/*f* noise free), a high sensitivity of 250 µC Gy_air_^−1^ cm^–2^, a signal drifting three orders of magnitude lower than all previous perovskite X-ray detectors^3–6,18^. The spatial resolution reaches 4.9 lp mm^−1^ at 20% modulation transfer function value. Taken together, our work showcases that Cs_2_AgBiBr_6_ wafer-based X-ray detectors are promising for next-generation X-ray imaging flat panels with features of stable output, low noise, high resolution, area scalability, and non-toxicity.

## Results

### Preparation of Cs_2_AgBiBr_6_ wafers

**Fig. 1:**
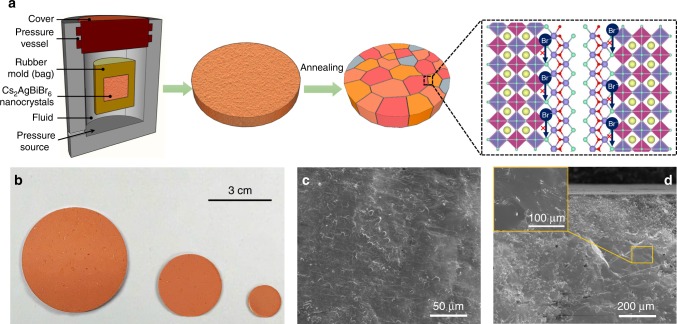
Isostatic-pressing method to prepare Cs_2_AgBiBr_6_ wafers. **a** Schematic illustration of the isostatic-pressing process, while Cs_2_AgBiBr_6_ powders were firstly modeled into a pie shape and then subsequently subjected to a pressure of 200 MPa through a hydraulic press, and the additional annealing process could enhance the crystallinity and grain growth. **b** As-prepared Cs_2_AgBiBr_6_ wafers with tunable sizes and the diameters are 5, 3, and 1 cm from left to right. **c** Top–down scanning electron microscopy (SEM) of the wafer. **d** Cross-sectional SEM image of the wafer and the inset is a higher resolution image, demonstrating the grain size is larger than 100 μm

Currently, it is still a great challenge to prepare large-area and millimeter-thick perovskite films for imaging applications. In a typical solution process, the inevitable solvent evaporation leaves behind amounts of pinholes within the thick film. The remaining pinholes severely hinder the charge transport and decrease the imaging resolutions, which is the case for previous MAPbI_3_-based X-ray imaging panels^6^. In addition, for Cs_2_AgBiBr_6_, the generally adopted solvent, HBr acid solution, is highly corrosive and narrows the selectable substrate for fabricating flat-panel detector arrays^19–21^. Thereby, we turn to an isostatic-pressing method for Cs_2_AgBiBr_6_ film fabrication^5^, which involves no solvent during the process and produces a pinhole-free and compact Cs_2_AgBiBr_6_ wafer, as shown in Fig. 1a.

If CsBr, AgBr, and BiBr_3_ were used as the precursors, we could sometimes observe the competitive phases of AgBr, CsAgBr_2_, Cs_2_AgBr_3_, and Cs_3_Ag_2_Br_9_ in the wafer especially for low-temperature annealing conditions, as shown in Supplementary Fig. 1. Hence, we directly utilized Cs_2_AgBiBr_6_ powders as the precursor to ensure phase purity. The powders were firstly ball-milled through Cs_2_AgBiBr_6_ single crystals, resulting in grain sizes between 100 nm and 1 μm (Supplementary Fig. 2). The use of Cs_2_AgBiBr_6_ single crystals as raw materials also helps guarantee the low defect states within the bulks. The powders were then modeled into a pie shape through a compressor and were subsequently subjected to a pressure of 200 MPa through a hydraulic press. The isotropic high pressure renders the wafer firmly flat and compact, leaving no pinholes within it. The pressing process is schematically shown in Fig. 1a. Here we prepared wafers with diameters of 5, 3, and 1 cm, producing an area of 19.62, 7.06, and 0.78 cm^2^, respectively, demonstrating the upscaling capability of this method (Fig. 1b). Further increase of the wafer size could be obtained by larger models. The thickness is set as 1 mm, which is enough for X-ray attenuation^7^. After pressing, the wafer was directly annealed at 350 °C for 20 h to promote grain growth. The fabricated wafer exhibits a pure Cs_2_AgBiBr_6_ phase without any other impurity (Supplementary Fig. 3). As shown in Fig. 1c, SEM shows a homogeneous and dense surface. The cross-sectional SEM shows a compact, pinhole-free composition (Fig. 1d). The grain size reaches hundreds of micrometers, as shown in the inset image as well as Supplementary Fig. 4, much larger than the powders (100 nm to 1 μm) in the precursor, which indicates substantial grain growth during isostatic pressing and thermal annealing process. The grain growth stems from the ionic nature of Cs_2_AgBiBr_6_ and crystal plasticity^5^. The increased grain size is beneficial for efficient carrier transport and charge collections.

### Structure analysis of Cs_2_AgBiBr_6_ wafers

Due to the polycrystalline nature, there are still amounts of residual grain boundaries within our Cs_2_AgBiBr_6_ wafer. According to the knowledge of Pb-based perovskites, abundant halide vacancies present at the grain boundaries, which could serve as the ionic migration channels and lead to severe baseline drift^22^. Similarly, our previous work demonstrates that bromide vacancies are also the major ionic migration channels for Cs_2_AgBiBr_6_^7^. We indeed observed serious baseline drift for the wafer (will present later). Thereby the grain boundaries within the wafer have to be passivated to decrease the bromide vacancies and suppress ionic migration. As we stated previously, we believe epitaxial passivation is an effective route, like CdS passivation for CdSe, AlN for GaN and MAPbBr_3_ for PbS^12–14^. Here we find BiOBr as a suitable choice as heteroepitaxial passivation layer for Cs_2_AgBiBr_6_. BiOBr could be easily prepared by hydrolysis of BiBr_3_ (also the precursor of Cs_2_AgBiBr_6_), guaranteeing the convenience and compatibility of the fabrication process.

Cs_2_AgBiBr_6_, the double perovskite, has a cubic structure, with the centers of metal bromide octahedron occupied by Bi^3+^ and Ag^+^. The lattice parameter of Cs_2_AgBiBr_6_ is *a* = *b* = *c* = 11.2499 Å. BiOBr crystallizes in the tetragonal matlockite structure (space group of P4/nmm), a layered structure with [Bi_2_O_2_] slabs interleaving by double slabs of halogen atoms^23^. The lattice parameters of BiOBr are *a* = *b* = 3.915 Å, *c* = 8.07 Å. Each Bi^3+^ could be seen as coordinating with four Br^−^ ions and four O^2−^ ions. In terms of structure compatibility, the arrangement of Br^−^ ions in BiOBr (001) plane is the same as those Br^−^ within the Cs_2_AgBiBr_6_ (001) plane. The distance between the neighboring two Br^−^ in BiOBr is equal to its lattice parameter along *x* or *y*-axis, i.e., 3.915 Å, while the distance between neighboring Br^−^ in Cs_2_AgBiBr_6_ is $\frac{{\sqrt 2}}{4}$ *a* = 3.977 Å. The lattice mismatch is then derived as 1.6%, and such small mismatch ensures the compatibility of these two species.

**Fig. 2:**
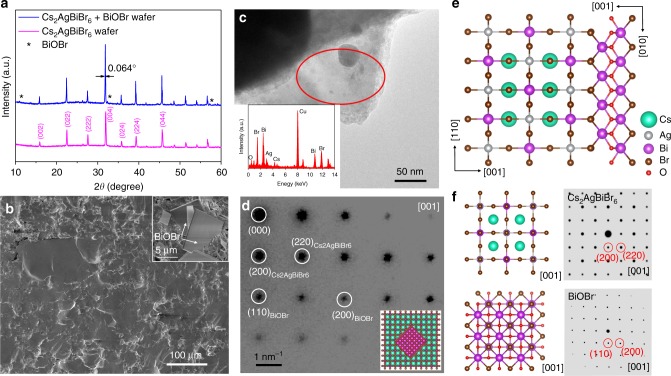
Structural determination of BiOBr-passivated Cs_2_AgBiBr_6_ wafer. **a** XRD spectra of the wafers with/without BiOBr passivation. **b** Cross-sectional SEM image of the BiOBr-passivated Cs_2_AgBiBr_6_ wafer, the inset is the top view. **c** TEM image of the selected region containing both BiOBr and Cs_2_AgBiBr_6_, the inset is the EDS result to verify the presence of Bi, O, Br, Cs, and Ag elements. **d** SAED pattern of the selected region in **c**, and the inset is the epitaxial growth model. The corresponding diffraction spots from Cs_2_AgBiBr_6_ and BiOBr are labeled accordingly, and the zone axis is [001]. **e** Epitaxial growth direction between BiOBr and Cs_2_AgBiBr_6_. **f** Crystal structure of Cs_2_AgBiBr_6_ and BiOBr viewed from [001] directions and the related diffraction pattern

Based on the above analysis, we added slightly more BiBr_3_ into the Cs_2_AgBiBr_6_ powders before isostatic pressing. The wafers were then thermally treated at 350 °C in the air atmosphere, and the moisture in the air could react with the excessive BiBr_3_ to form BiOBr. It should be noted that the melting point of BiBr_3_, CsBr, AgBr is 218, 636, 432 °C, respectively. The thermal treatment at 350 °C could melt BiBr_3_ and the melted ions are beneficial for defects repairing within the films. The mass ratio of BiBr_3_/Cs_2_AgBiBr_6_ in the precursor was optimized as 1%, while the further increase would block the carrier transport. As shown in Fig. 2a, we could clearly see the characteristic diffraction peaks of BiOBr, and there is no BiBr_3_ remaining in the products, verifying the complete reaction of BiBr_3_ after thermal annealing. Besides, the full width half maximum (FWHM) corresponding to (004) diffraction reached 0.064°, which is nearly identical to the 0.061° of Cs_2_AgBiBr_6_ single crystal^7^, confirming its high crystallinity. The representative cross-sectional SEM image of the wafer is shown in Fig. 2b, and the grain size is 30 to 100 μm after BiOBr introduction, similar to the wafer without BiOBr passivation (Supplementary Fig. 4f). The sheet-structured BiOBr could be clearly observed on the surface of the wafer (inset of Fig. 2b), further confirming the presence of BiOBr.

TEM characterization is used to verify the heteroepitaxial growth between BiOBr and Cs_2_AgBiBr_6_. It is difficult to record images at high-resolution mode due to the easy decomposition of Cs_2_AgBiBr_6_ under strong electron beam radiation^24^. Thereby the study was conducted at a low accelerating voltage, and the composition and structure were determined with energy disperse spectroscopy (EDS) and selected area electron diffraction (SAED). As demonstrated in Fig. 2c, we located an area containing Cs, Ag, Bi, Br, O, Cu atoms, while Cs, Ag, Bi, and Br were from Cs_2_AgBiBr_6_, and Bi, Br, O came from BiOBr, Cu came from copper grids. The SAED shows that the selected area contains only one set of diffraction spots (Fig. 2d), demonstrating the single crystal nature. Based on the diffraction spots, the lattice fringes could be calculated as 3.94 Å and 5.46 Å, which is consistent with inter-distance (220) and (200) planes of Cs_2_AgBiBr_6_, and above values are just twice of the inter-distance of (200) and (110) planes in BiOBr. The inset in Fig. 2d shows the epitaxial growth model between Cs_2_AgBiBr_6_ and BiOBr crystals. We also observed the similar composition and SAED patterns in several other regions, verifying the epitaxial relationship between Cs_2_AgBiBr_6_ and BiOBr (Supplementary Fig. 5). The crystal structures of Cs_2_AgBiBr_6_ and BiOBr are depicted in Fig. 2e for structure compatibility comparison. As stated previously, Cs_2_AgBiBr_6_ can connect with BiOBr along [001] direction by sharing the interface Br^−^ ions. With the zone axis as [001], the simulated SAED patterns of Cs_2_AgBiBr_6_ and BiOBr also totally echo with the measured result (Fig. 2f). The crystal structure analysis and SAED results together identified the orientation relationships between Cs_2_AgBiBr_6_ and BiOBr as {001}_Cs2AgBiBr6_‖{001}_BiOBr_, which is further supported by the observed epitaxial growth of Cs_2_AgBiBr_6_ microcrystals onto BiOBr sheets (Supplementary Fig. 6).

### Optical and electrical properties of Cs_2_AgBiBr_6_ wafers

**Fig. 3:**
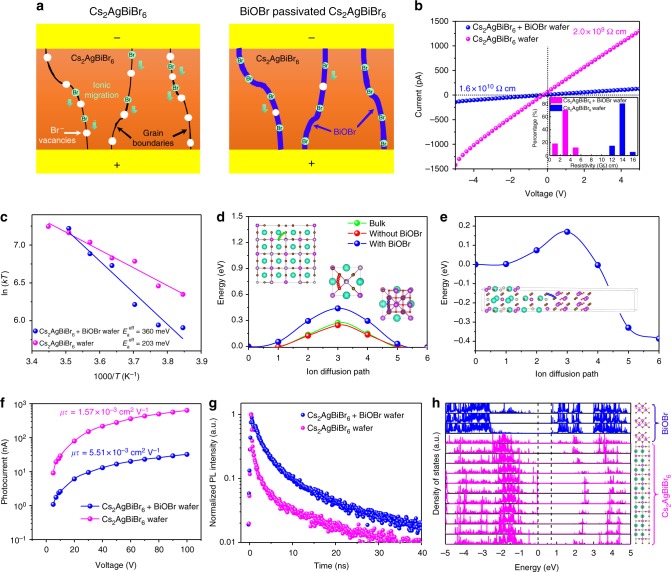
Optical and electrical properties of Cs_2_AgBiBr_6_ wafers with/without BiOBr passivation. **a** Schematic illustration of the suppressed ionic migrations by BiOBr passivation. **b** The resistivity of the wafers and the inset is the statistical result. **c** Arrhenius plots of the temperature dependence of *kT* versus 1000/*T*, while *k* is the ionic migration rate (s^−1^) and is proportional to the ionic conductivity. The fitting curve gives the activation energy of ionic migration, also known as the ion diffusion barriers. **d** Calculated energy profile along the ionic migration path for *V*_Br_ (Br^−^ vacancies) in bulk Cs_2_AgBiBr_6_, in-plane migration for surface of Cs_2_AgBiBr_6_ without and with BiOBr passivation. Inset: migration path of *V*_Br_ with the Br^−^ highlighted in green (bulk), red (surface without BiOBr passivation), and blue (surface with BiOBr passivation) color, respectively. **e** Calculated energy profile of the out-of-plane ionic migration path for the *V*_Br_ in BiOBr-passivated Cs_2_AgBiBr_6_. Inset: Migration path of V_Br_ with the Br^−^ highlighted in green, and the *V*_Br_ migration direction is from BiOBr to Cs_2_AgBiBr_6_. **f** Bias-dependent photoconductivity of the wafers and the derived *μτ* value. **g** The time-dependent photoluminescence of the wafers. **h** Layered decomposed density of states for Cs_2_AgBiBr_6_/BiOBr heterostructures

From the above analysis, we conclude that BiOBr could indeed epitaxially grow onto Cs_2_AgBiBr_6_ crystals, and the presence of BiOBr is expected to suppress the surface defects at Cs_2_AgBiBr_6_ grain boundaries. The schematic model is shown in Fig. 3a. Br^−^ vacancies (*V*_Br_) are previously found as the main species accounting for ionic migration in Cs_2_AgBiBr_6_^7^. Here the presence of BiOBr could supply Br^−^ to suppress most of Br^−^ vacancies, thereby reducing the ionic migration channels and decreasing the ionic conductivity. To verify the influence of BiOBr passivation layer, we measured the resistivity of the wafers. As shown in Fig. 3b, after introducing BiOBr epitaxial layer, the wafer resistivity increased from 2.0 × 10^9^ to 1.6 × 10^10^ Ω cm. The average resistivity of multiple Cs_2_AgBiBr_6_ wafers with BiOBr (1.4 × 10^10^ Ω cm) is about five times higher than that of pristine Cs_2_AgBiBr_6_ (3.0 × 10^9^ Ω cm). The resistivity enhancement is caused by the suppressed ionic conductivity and defects, as well as the insulating effect of BiOBr for electric conductivity. The higher resistivity is beneficial for achieving a declined dark current and thus abated noise current for the fabricated detectors.

To quantitatively characterize the ionic migration, we adopt the previously established method to directly extract the ion diffusion barrier^7,25^. The measurement details are shown in Supplementary Fig. 7 and Supplementary Note 1. In a nutshell, we exerted an exterior voltage to the crystal to direct the ionic diffusion, and the movable ions could accumulate at the border between Cs_2_AgBiBr_6_ and Au electrode. Upon turning off the voltage, the vacant positions of ion expeditiously migrated backward due to the concentration grade, resulting in an opposing current. The decay in the opposing current can utterly manifest the dynamics of the ion motion. Hence, the decay rate (*k* = *τ*^−1^), obtained by mono-exponential fitting, embodies the ion motion behaviors and is in proportion to the ion conductivity (*σ*_ion_). By measuring the temperature-dependent current decays, the ion diffusion barrier can be obtained by fitting fitting ln(*kT*) versus 1/*T*. Figure 3c shows that *E*_a_^eff^ for BiOBr-passivated Cs_2_AgBiBr_6_ wafer is 360 meV, much higher than that of the pure Cs_2_AgBiBr_6_ wafer (203 meV) and even slightly higher than Cs_2_AgBiBr_6_ single crystals (348 meV), indicating the defects at the grain boundaries have been perfectly suppressed by BiOBr passivation^7,22^. Besides, the ion diffusion barrier of MAPbI_3_ and MAPbBr_3_ is only 134 and 127 meV according to the previous studies^26^. The much higher diffusion barrier of BiOBr-passivated Cs_2_AgBiBr_6_ wafer demonstrates its promising potential for ionic migration-free devices.

We also applied first-principle calculations to study the influence of BiOBr on ionic migrations of Cs_2_AgBiBr_6_. The detailed calculation processes are included in supplemental materials (Supplementary Figs. 8 to 18). In bulk Cs_2_AgBiBr_6_, the diffusion barrier of *V*_Br_ is calculated as 0.30 eV, consistent with our previous result (0.33 eV)^7^. On the bare surface of Cs_2_AgBiBr_6_ (Fig. 3d), the diffusion barrier of *V*_Br_ was slightly reduced to 0.25 eV, indicating that the Br vacancy migration is enhanced at the non-passivated Cs_2_AgBiBr_6_ surface. Nevertheless, after passivation by BiOBr layers, the diffusion of *V*_Br_ on the Cs_2_AgBiBr_6_ surface (more precisely, at the Cs_2_AgBiBr_6_ side of the Cs_2_AgBiBr_6_/BiOBr interface) is greatly suppressed, as its migration barrier is increased to 0.44 eV. For out-of-plane diffusion, we also calculated the barriers for V_Br_ moving out of Cs_2_AgBiBr_6_ as 0.56 eV, in the same Cs_2_AgBiBr_6_/BiOBr interface model (Fig. 3e). In addition, the *V*_Br_ migration barrier of BiOBr itself is 0.99 eV, which is too high to allow any migration (Supplementary Fig. 16). Clearly, the presence of BiOBr can cut off *V*_Br_ migration path and mitigate the ionic migrations, in addition to the passivation of defects at grain boundaries.

The *μτ* products have also been measured, while *μ* is the mobility of carrier and τ is the lifetime. The *μτ* product represents the distance that the carrier can transit under specific electric field strength. We employed a modified Hecht equation for fitting the photoconductivity curve to extract *μτ* value^27,28^. As exhibited in Fig. 3f, Cs_2_AgBiBr_6_ wafer without BiOBr passivation held a *μτ* product of 1.57 × 10^–3^ cm^2^ V^−1^. After BiOBr introduction, the *μτ* product boosted to 5.51 × 10^–3^ cm^2^ V^−1^. The increased *μτ* product, which can upgrade the extraction efficacy of photo-induced carriers, is due to the suppressed defects and increased carrier lifetime, the latter of which is verified by photoluminescence lifetime measurement. As shown in Fig. 3g, three processes were exhibited via the photoluminescence (PL) decay of pristine Cs_2_AgBiBr_6_ wafer: short (0.81 ns), intermediate (4.79 ns), and long (35.01 ns) lifetime components. The short and intermediate lifetime originate from surface-state emission, and the long lifetime is from bulk phase^19^. By introducing BiOBr, both defects at grain boundaries and within bulks could be suppressed and the photoluminescence lifetime was prolonged, resulting in the short-lifetime component as 2.29 ns, the intermediate lifetime as 10.99 ns, and the long lifetime as 63.05 ns.

In addition, as we calculated for the density of states of the heteroepitaxial interface, there is no additional density of states introduced within the band gaps, confirming the good passivation effect of BiOBr (Fig. 3h). Also, the conduction band edge of BiOBr falls within the band gap of Cs_2_AgBiBr_6_. Cs_2_AgBiBr_6_ and BiOBr form a Type-II heterojunction (Supplementary Fig. 19), and the high carrier mobility of BiOBr guarantees the effective charge extraction^23^. The presence of type-II heterojunction and potential charge transfer could also contribute to the observed longer PL lifetime. The low ionic migration and good carrier mobility within BiOBr make the heterojunction structure highly resistant toward ion migration and conductive for carrier transport, which is supreme for X-ray detection applications.

### X-ray detection and imaging

We now assemble the X-ray detector, where Au, Cs_2_AgBiBr_6_ wafer and Au vertically stack together. The device was illuminated by X-ray source employing a tungsten anode, the largest X-ray energy of which is 50 keV and the intensity climax is at 30 keV. We then calibrated the dose rate by a Radcal ion chamber dosimeter. It is noteworthy that the air ionization between cathode and anode electrodes could also contribute to the photocurrent. We subject the detectors into a vacuum trap to exclude the influence of air ionization.

**Fig. 4:**
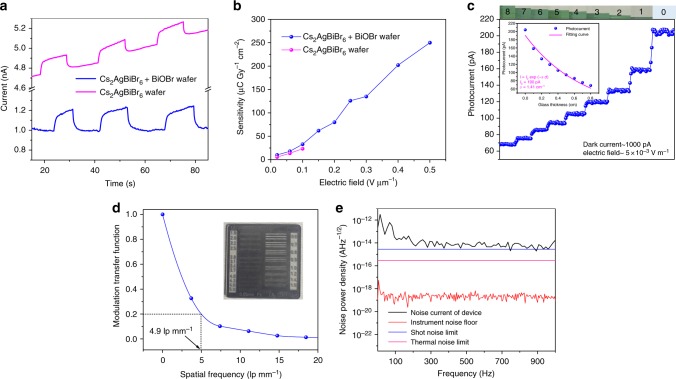
Performance of Cs_2_AgBiBr_6_ wafer X-ray detector. **a** Device response to X-rays (138.7 μGy_air_ s^−1^) under an electric field of 0.1 V μm^−1^. **b** X-ray sensitivity under different electric fields. **c** The response of the detectors toward stacked ITO glass coverslips. **d** Modulation transfer function for the fabricated detector and the inset is the line pair card for edge spread function measurement. **e** Measured dark current noise at various frequencies

The photocurrent response under an electric field of 0.1 V μm^−1^ for Cs_2_AgBiBr_6_ wafer-based X-ray detectors is present in Fig. 4a and the dose rate is 138.7 μGy_air_ s^−1^. The wafer passivated with BiOBr has a stable baseline and the dark current is about 1 nA. In contrast, the dark current keeps increasing for the pure Cs_2_AgBiBr_6_ wafer, and thus the signal reading becomes inaccurate. We also compare our results with previously reported perovskite-based X-ray and γ-ray detectors in terms of the applied electric field and dark current drift (Supplementary Table 1 and Supplementary Fig. 20). Clearly, the dark current drift of BiOBr-passivated Cs_2_AgBiBr_6_ wafer is 7.4 × 10^–5^ nA cm^−1^ s^−1^ V^−1^ under an electrical field of 0.5 V μm^−1^, one order of magnitude lower than the best value of Pb-based perovskites (1.4 × 10^–4^ nA cm^−1^ s^−1^ V^−1^)^29^ under a 50 times weaker electric field. The stabilization effect of BiOBr was due to the defect passivation and decreased ionic migrations. We then only calculate the sensitivity for BiOBr-passivated wafers. The measured photocurrent density scales linearly with the X-ray dose rate (Supplementary Fig. 21). The dose used here is the entrance dose in air and the electric field is 0.1 V μm^−1^. A sensitivity of 32 µC Gy_air_^−1^ cm^–2^ was derived for the wafer. The photocurrent responses under different electric field strengths and dose rates were also recorded (Supplementary Fig. 22). To compare the temporal rise behavior for the two devices, we conducted baseline subtraction for Cs_2_AgBiBr_6_ wafer in Fig. 4a. Then the response curves for Cs_2_AgBiBr_6_ and Cs_2_AgBiBr_6_ + BiOBr wafer were drawn together, showing similar temporal rise behavior (Supplementary Fig. 23).

As the ionic migration has been successfully suppressed by BiOBr passivation, then we record the detector sensitivity with the electric field ranging from 0.02 to 0.5 V µm^−1^. As shown in Fig. 4b, the sensitivity linearly increased from 10 to 250 µC Gy_air_^−1^ cm^–2^, which is twelve times higher than α-Se X-ray detectors (20 µC Gy_air_^−1^ cm^–2^) operating at a much higher field of 10 V µm^−1^^30^. The sensitivity of our polycrystalline wafer is even higher than the Cs_2_AgBiBr_6_ single crystal-based device (105 µC Gy_air_^−1^ cm^–2^ at 0.025 V µm^−1^). Previous Pb-based perovskites typically exhibit severe ionic migrations, excluding the use of large bias. We want to emphasize that high bias voltage is highly important for radiation detections, as it reduces the carrier transit time between electrodes, decreases the carrier diffusion length perpendicular to the electric field direction, and thereby prohibits the signal crosstalk between different pixels for imaging applications^31^. The imaging contrast result was shown in Fig. 4c. Stacking ITO glasses serve as X-ray attenuators, and the numbers represent the layers. The photocurrent decreases exponentially with the increase of stacking layers. We could derive the attenuation coefficient as 1.41 cm^−1^ for ITO glasses, which is consistent with the theoretically calculated attenuation coefficient (1.42 cm^−1^).

Detection limit is a very important parameter to evaluate the device performance. By using the IUPAC standard of signal-to-noise ratio of 3, the detection limit of Cs_2_AgBiBr_6_ + BiOBr wafer is measured as 95.3 nGy_air_ s^−1^, which is slightly worse than our previous Cs_2_AgBiBr_6_ single crystal detector (59.7 nGy_air_ s^−1^) but much lower than 571.3 nGy_air_ s^−1^ of Cs_2_AgBiBr_6_ wafer (Supplementary Fig. 24).

Then we investigated the imaging capability of the fabricated detectors. The well-acknowledged slanted-edge method was adopted for the modulation transfer function (MTF) measurement, i.e., imaging the sharp edge of the line pair card with a single pixel^32^. The details are shown in Supplementary Fig. 25 and Supplementary Note 2. The pixel size was 200 μm × 200 μm, and the line pair card was fixed on an *x*–*y* scanning stage and transport along the direction of y axis for acquiring the edge imaging. The response for the edge was recorded as the edge spread function (ESF), and the ESF was then differentiated to produce the line spread function (LSF), as shown in Supplementary Fig. 25. The MTF could be derived by applying a fast Fourier transformation to the LSF. The detailed calculation process of MTF is shown in supporting information. The resulting MTF is presented in Fig. 4d, and the resolution is about 4.9 lp mm^−1^ at 20% MTF value, which is higher than previous MAPbI_3_-based imaging system (3.1 lp mm^−1^)^6^. We remark that the resolution of the detector is related to the pixel size, pinholes within the film and signal crosstalk. Our compact wafer and the use of high electric field all contribute to the enhanced spatial resolution.

For imaging application, noise is very important and hence the noise spectrum was also evaluated through a spectrum analyzer. The BiOBr-passivated Cs_2_AgBiBr_6_ wafers are dominated by 1/*f* noise in the low-frequency region, which is probably caused by the presence of surface traps on the wafer (Supplementary Fig. 26). Here we introduced polyimide (PI) as an interface layer between perovskite and gold electrodes. It is found that the resulted noise power density (3.62 × 10^–15^ A Hz^−1/2^) is independent on frequency ranging from 1 to 1000 Hz with basically preserved device sensitivity (Supplementary Fig. 27), which may result from the full passivation of surface traps on the wafer by a thin PI layer (Supplementary Fig. 28). The shot noise and thermal noise were calculated as 2.83 × 10^–15^ and 2.88 × 10^–16^ A Hz^−1/2^, respectively. The total white noise was then calculated as 3.12 × 10^–15^ A Hz^−1/2^ by including shot noise and thermal noise, close to the measured result (Fig. 4e). The suppressed 1/*f* noise enables the use of low modulation frequency (1 or 30 frame per second) for signal reading.

**Fig. 5:**
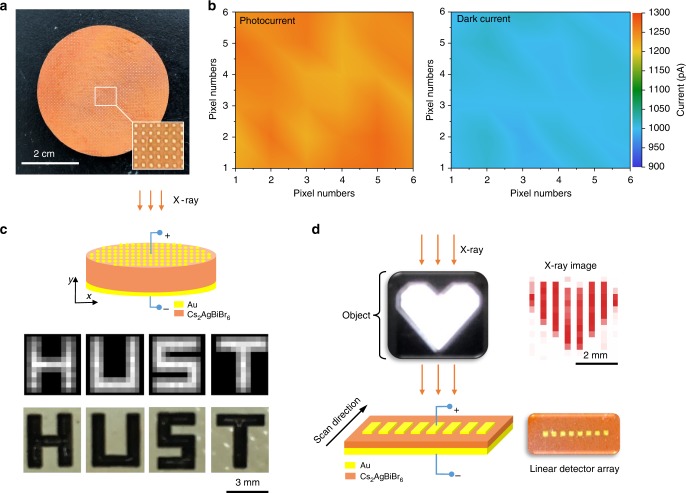
Imaging applications of Cs_2_AgBiBr_6_ wafer X-ray detector. **a** The fabricated multi-pixel wafer-based detector. **b** Mapping of photocurrent (left) and dark current (right) for the wafer with 6 × 6 pixels as the region of interest. **c** The Schematic illustration of the imaging process, and the X-ray image (top) and the optical image (bottom) of ‘HUST’ symbol. **d** Optical image and X-ray image of the heart-shaped logo obtained by the linear detector array, the dose rate for imaging is 138 μGy_air_ s^−1^, and the scanning mode, as well as the linear detector array is shown at the bottom

The uniformity of dark current and photocurrent is decisive on imaging quality. According to the acknowledged regulation, X-ray image should not have obvious artefacts, and the ratio of the standard deviation to the mean signal should be <10%^33^. Herein the pixel uniformity was measured within a 6 × 6 pixel array selected from the fabricated array in Fig. 5a, with the average value of dark current as 1003 ± 7 pA, and photocurrent as 1243 ± 15 pA, as shown in Fig. 5b. The ratio of standard deviation to the mean signal is calculated as 0.7% for dark current and 1.2% for photocurrent, which are an order of magnitude lower than the standard regulation. To further simulate the imaging application of the wafer-based detector, a planar array was fabricated. The X-rays were detected by the detector after traveling through an iron ‘HUST’-shaped logo. The symbol ‘HUST’ was scanned and the recorded image is shown in Fig. 5c. We also assembled an eight-pixel linear array detector with a pixel size of 0.8 mm and a pitch of 0.8 mm (Fig. 5d). The pixels were connected to the electrometer. By linearly scanning in one direction, the heart-shaped logo could be easily distinguished. The spatial resolution could be further enhanced by integrating the fabricated wafers onto thin film transistor arrays with smaller pixel sizes (50 to 150 μm).

## Discussion

One promising application of perovskite X-ray detectors is static/dynamic digital radiography (SDR/DDR), which requires the photoelectric conversion efficiency larger than 30 e^−^keV^−1^, and frame rates of 1 frame per second (FPS) for SDR or 30 FPS for DDR^34^. These are within the capability of perovskite X-ray detectors. Baseline drift, also well known as ‘polarization’, is a common difficulty for all kinds of direct X-ray detectors including *α*-Se, CdZnTe, and perovskites. Here the polarization issue has been resolved by the presented heteroepitaxial passivation method. One unaddressed issue for all reported perovskite X-ray detectors is the required dark current density lower than 10^–10^ A cm^−2^^35^. This value is derived from the minimal dynamic range constraint: *Q*_dark_ = *Q*_signal_, *Q*_dark_ is *J*_dark_ × A × Δ*t*, and *Q*_signal_ is *eE*_photon_/(2.8*E*_g_ + 0.5 eV) from one X-ray photon, where *A* is the pixel area (typically 100 μm × 100 μm), Δ*t* is charge read out time, *E*_photon_ is photon energy of X-ray (10 to 50 keV). P-N or Schottky junctions could be introduced to suppress the dark current. Moreover, charge−sensitive preamplifiers, with the characters of high gain and ultra-low noise (lower than 100 e^−^), are suitable back-end electronic components to further enhance the detector dynamic range and signal quality.

In conclusion, we utilized an isostatic-pressing method to prepare Cs_2_AgBiBr_6_ wafers with tunable sizes, achieving non-toxic, large-area and high-performance X-ray detectors for imaging. The epitaxial growth of BiOBr onto Cs_2_AgBiBr_6_ enables the perfect grain boundary passivation and helps suppress ionic migrations to single crystal level. The signal drifting of the detector is three orders of magnitude lower than all previous perovskite X-ray detectors. Device sensitivity reaches 250 µC Gy _air_^−1^ cm^–2^ at 0.5 V μm^−1^ bias, 12 times higher than α-Se detectors. The spatial resolution is about 4.9 lp mm^−1^ at an MTF value of 0.2. Thanks to the high uniformity and low noise current, we built 6 × 6 pixel arrays on our Cs_2_AgBiBr_6_ wafer and explored their imaging application. Further optimizing the pixel configuration and assembling Cs_2_AgBiBr_6_ wafers onto thin film transistor arrays or CMOS chips would push them toward practical imaging applications. Overall, the combined features of low-cost fabrication, area scalability, non-toxicity, stable output and ultra-low noise strengthen the competitiveness of Cs_2_AgBiBr_6_-based X-ray detectors as next-generation X-ray imaging flat panels.

## Methods

### Materials

CsBr (99.9%) was purchased from Aladdin Reagent Ltd. AgBr (99.9%) and BiBr_3_ (99%) were from Sigma-Aldrich. Hydrobromic acid (HBr, 40% wt/wt aq. sol.) and 1-methyl-2-pyrrolidone (NMP, 99%) was purchased from Sinopharm. *p*-Phenylenediamine (97%) was from Macklin, 4,4′-biphthalic anhydride (BPDA, 99%) from J&K Scientific. All reagents were used as received.

### Cs_2_AgBiBr_6_ single crystals

Cs_2_AgBiBr_6_ crystals were grown using an improved inverse temperature crystallization method. Specifically, CsBr (0.002 mol, 0.426 g), BiBr_3_ (0.001 mol, 0.449 g), and AgBr (0.001 mol, 0.188 g) were added into HBr solution (12.5 mL) within a glass container. The container was firmly covered, heated to 110 °C to entirely dissolve the precursors. The solution was then cooled to 80 °C to promote crystal growth.

### Preparation of Cs_2_AgBiBr_6_ wafer

Cs_2_AgBiBr_6_ crystals were firstly ball-milled for about five hours to achieve uniform Cs_2_AgBiBr_6_ powders. The powders were then mounted into a pie shape mold through a compressor, and subsequently were subjected to a pressure of 200 MPa through a hydraulic press (LDJ-100/320-300). By changing the mold, the wafer size could be readily modulated, and the targeting thickness could be obtained by adjusting the pressing pressure and precursor loading amount. The wafer was then annealed on the hot plate at 200 to 350 °C for 4 to 20 h in air for further crystallization, the optimized condition was 350 °C for 20 h.

### Device fabrication

A 1-mm-thick Cs_2_AgBiBr_6_ wafer X-ray detector was employed for device assembly, where Au/Cs_2_AgBiBr_6_ wafer/Au stacked vertically. The Au electrodes (about 80 nm) were thermally evaporated. To suppress 1/*f* noise, we introduced polyimide (PI) as an interface layer between perovskite and gold electrodes. For 1% weight ratio of PI precursor preparation, 0.0336 g p-phenylenediamine and 0.0956 g BPDA were added into 12.6 mL NMP, and the precursor was blended for 8 h at room temperature and consecutively at 40 °C for 12 h. Then the precursor was drop-coated onto the surface of Cs_2_AgBiBr_6_ wafer, and then the solution was cured for 10 to 30 min on a 120 °C hot plate.

### Material characterization

The crystal structure and phase purity of Cs_2_AgBiBr_6_ wafer were identified by X-ray diffraction (XRD, Philips, X pert pro MRD, Cu Kα radiation, *λ* = 1.54178 Å). Morphology of Cs_2_AgBiBr_6_ wafer was studied by SEM (FEI Nova NanoSEM450, without Pt coating). TEM was acquired through Tecnai-G^2^ 20U-TWIN. Time-resolved photoluminescence was tested at 630 nm under a 478 nm light pulse as excitation from the HORIBA Scientific DeltaPro fluorimeter.

### Detector performance measurement and X-ray imaging

X-ray tube with tungsten anode (HAMAMATSU L9421–02) was used as the source. The X-ray focal spot size is 5 μm. A Keithley 6517B provided the bias voltage and recorded the response current. For noise currents measurement, 5 V bias was exerted onto the device by low noise current amplifier (SR570) and the output was coupled to lock-in amplifier (SR850). SR570 worked in high bandwidth mode (High BW) without any filter. The X-ray source was under a constant 50 kV voltage. The current was changed from 1 to 160 μA. Al foil (2 mm thick) was used as the attenuator between the X-ray tube and Cs_2_AgBiBr_6_ wafer. The dose rate has been evaluated with a Radcal ion chamber (model: 10 × 6–180) dosimeter. The measurement was operated in the dark and subjecting the device in vacuum to avoid the influence from visible light and air ionization. For acquiring X-ray imaging, the object was adhered on a *x*–*y* scanning stage (Zolix PSA200–11-X), and we controlled the object to transport in the directions along x and y axis.

### Computational methods

First-principle calculations were carried out using density functional theory, as implemented with the Vienna *Ab initio* simulation package (VASP)^36^. The projector augmented-wave (PAW) method was used, with a fixed 500 eV plane-wave kinetic energy cutoff^37^. The electrons regarded as valence were: 5*s*, 5*p*, and 6*s* for Cs, 4*d* and 5*s* for Ag, 6*s* and 6*p* for Bi, 2*s* and 2*p* for O, and 4*s* and 4*p* for Br, while core electrons were estimated by PAW pseudopotentials. The generalized gradient approximation (GGA) was used for the exchange-correlation energy, within the Perdew–Burke–Ernzerhof (PBE) functional^38^. Self-consistent calculations were carried out with a tight energy convergence criterion of 10^–6^ eV difference between two consecutive electronic steps. The DFT-D3 correction^39^ was employed in our calculations to illustrate the Van der Waals interaction in the hetero structure models. For geometric optimization, all configurations were relaxed until the Hellmann–Feynman forces were below 0.01 eV Å^−1^, and for bulk materials the stress is kept minimum below 100 MPa in any direction. In interface/surface-containing supercells, the stress in each direction was kept below 1.2 GPa, and a vacuum layer of 15 Å along the *z* direction was introduced to minimize the artificial interactions between adjacent slabs.

For electronic structure calculations, the spin–orbit coupling (SOC) effect is considered for all models. In order to conquer the notable band gap issue of DFT, we utilized the GGA-1/2 self-energy correction method^40^, based upon a GGA + U ground state. We used a Hubbard U of 5 eV for the 4*d* orbital of Ag^41^, and the optimal self-energy cutoff radii in GGA-1/2 were found to be 3.3 bohr for Br in Cs_2_AgBiBr_6_, 3.8 bohr for Br in BiOBr, and 2.5 bohr for O in BiOBr. The band alignment comparison with two kinds of termination states for Cs_2_AgBiBr_6_ was documented in supporting information (Supplementary Fig. 18)^42,43^.

The ion diffusion barriers were investigated by using the climbing image nudged elastic band (CI-NEB) method^44^. In the CI-NEB calculation, the Hellman–Feynman forces on each atom were smaller than 0.05 eV Å^−1^ and the energy convergence with the energy difference was below 10^–5^ eV between two consecutive self-consistent steps. Moreover, we also performed calculations involving dipole corrections. As shown in Supplementary Figs. 11–15, after introducing the dipole correction, the calculated ion diffusion barriers show extremely tiny difference, even within the systematic computational error. This implies that dipole correction is not required for these calculations.

## Supplementary information

Supplementary Information

## Data Availability

The data that support the findings of this study are available from the corresponding author on request.
