# Supplementary material for: Heteroepitaxial passivation of Cs2AgBiBr6 wafers with suppressed ionic migration for X-ray imaging
Source: Nat Commun. 2019 Apr 30;10:1989. doi: 10.1038/s41467-019-09968-3 (PMC6491557; doi:10.1038/s41467-019-09968-3)
Supplement: Supplementary file 1 — Supplementary Information [file 41467_2019_9968_MOESM1_ESM.doc]

**Supplementary materials online**

**Heteroepitaxial passivation of Cs_2_AgBiBr_6_ wafers with suppressed ionic migration for**

**X-ray imaging**

Bo Yang^1,2†^, Weicheng Pan^1,2†^, Haodi Wu^1,2†^, Guangda Niu^1,2*^, Jun-Hui Yuan^2^, Kan-Hao Xue^2^, Lixiao Yin^1,2^, Xinyuan Du^1,2^, Xiang-Shui Miao^1,2^, Xiaoquan Yang^1^, Qingguo Xie^3^, Jiang Tang^1,2*^

^1^Wuhan National Laboratory for Optoelectronics (WNLO), and ^2^School of Optical and Electronic Information, Huazhong University of Science and Technology (HUST), Wuhan, 430074, China

^3^College of Life Science and Technology, Huazhong University of Science and Technology, Wuhan 430074, China.

^†^These authors contributed equally to this work.

Correspondence and requests for materials should be addressed to G. N. and J. T. (E-mail: guangda_niu@hust.edu.cn; jtang@mail.hust.edu.cn)

**Supplementary Figures**


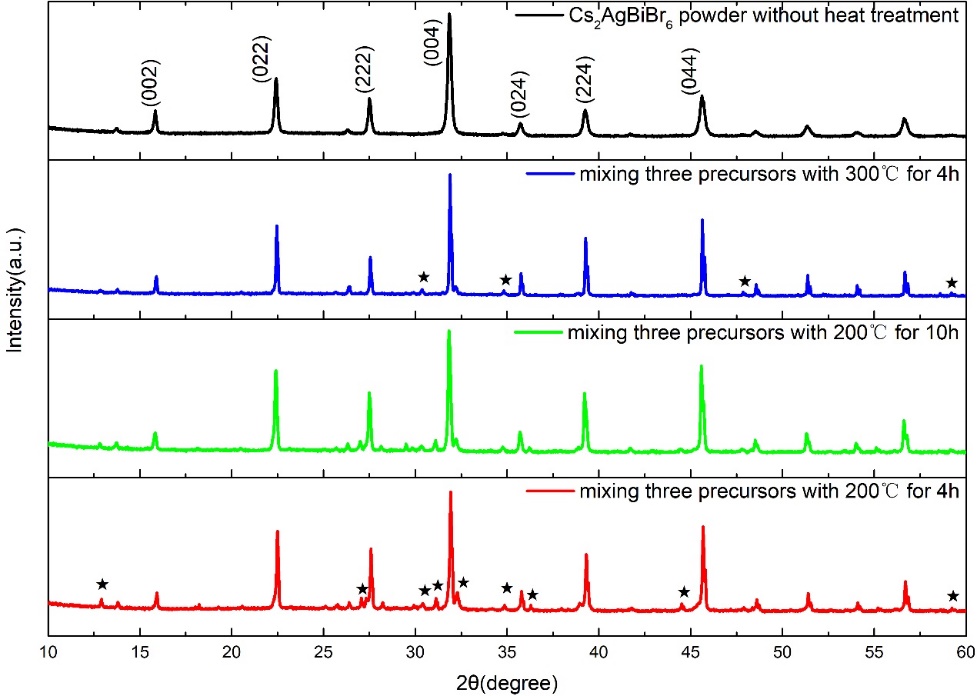


**Supplementary Figure 1.** XRD patterns of Cs_2_AgBiBr_6_ wafers prepared through raw materials of CsBr, AgBr and BiBr_3_ (peaks labeled by ★ represent the competitive phases of AgBr, CsAgBr_2_, Cs_2_AgBr_3_ and Cs_3_Ag_2_Br_9_).


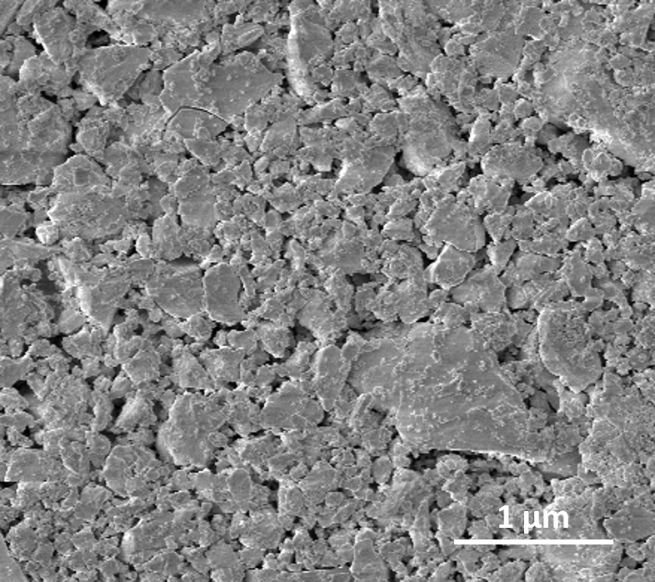


**Supplementary Figure 2.** SEM image of Cs_2_AgBiBr_6_ powders obtained by ball-milling Cs_2_AgBiBr_6_ single crystals. The powder size ranges from 100 nm to 1 μm.


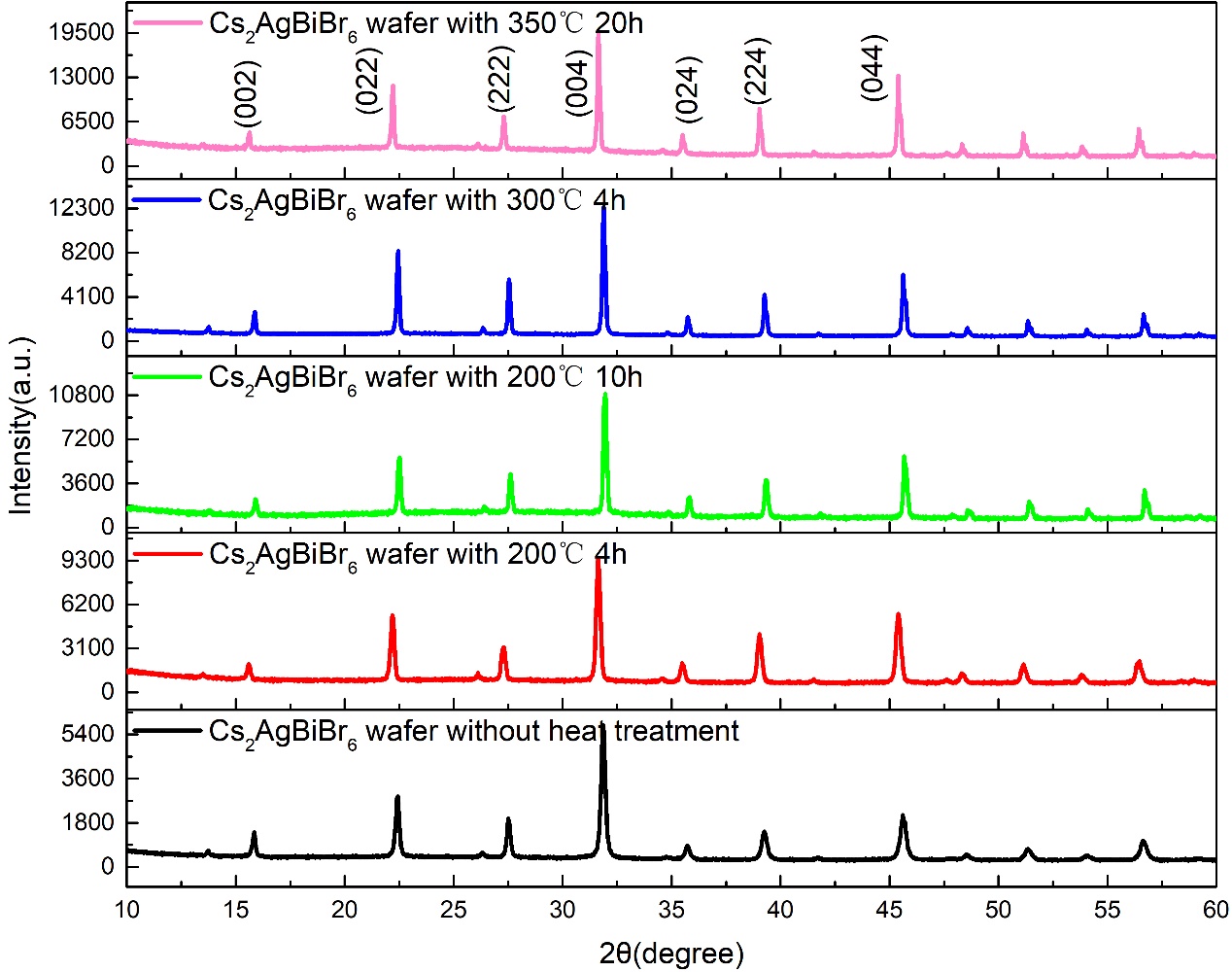


**Supplementary Figure 3.** XRD patterns of Cs_2_AgBiBr_6_ wafers with different heat treatments. The results indicate that all samples have pure Cs_2_AgBiBr_6_ phase, and the XRD peaks become stronger and narrower with increasing annealing temperature.


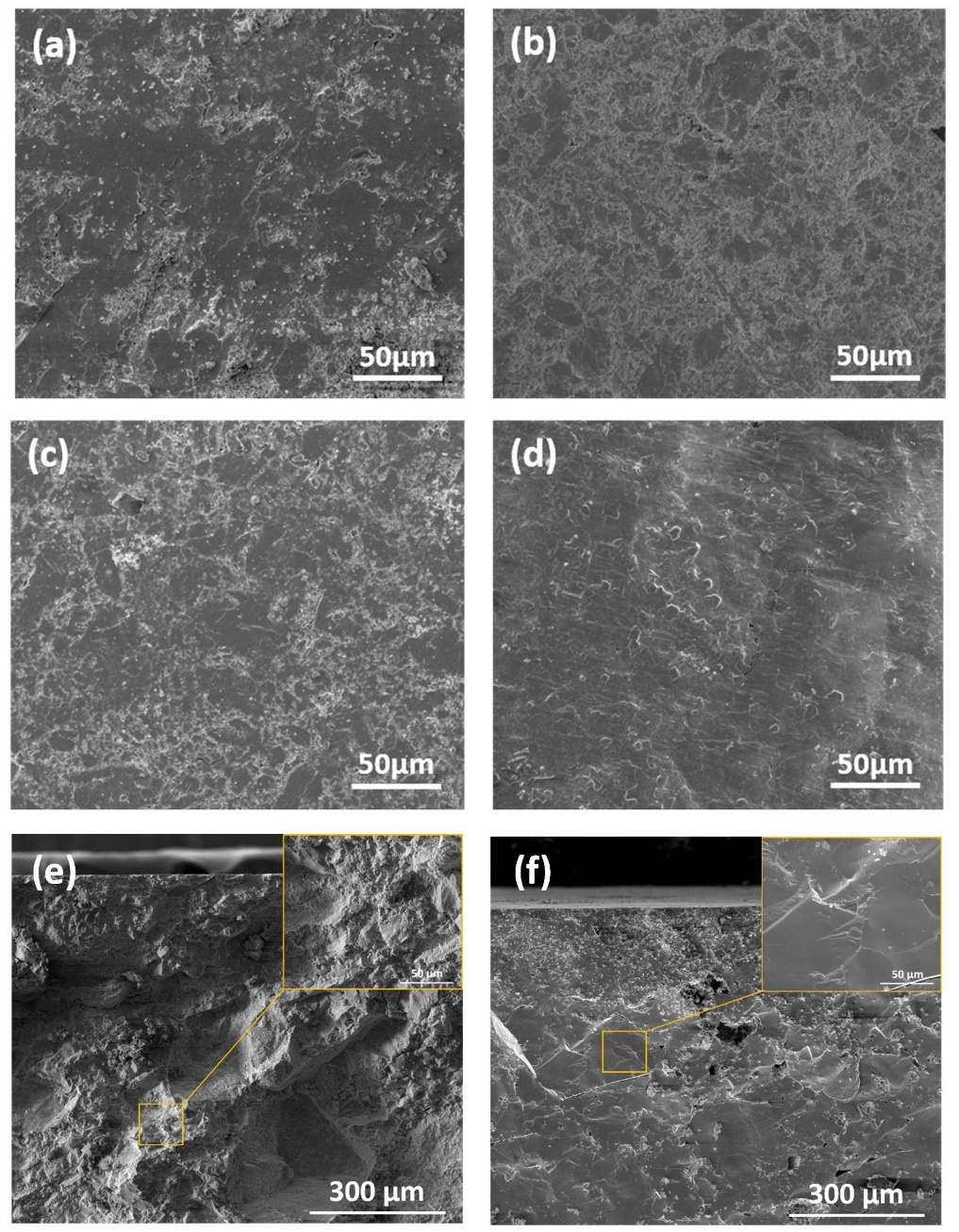


**Supplementary Figure 4.** Top-down SEM images of Cs_2_AgBiBr_6_ wafers from different treatment conditions, (a) 200 ℃ for 4 h, (b) 200 ℃ for 10 h, (c)300 ℃ for 4 h, (d) 350 ℃ for 20 h; Cross-sectional SEM images of Cs_2_AgBiBr_6_ wafers from different treatment conditions, (e) 200 ℃ for 4 h and (f) 350 ℃ for 20 h, and the insets are high resolution images.

**
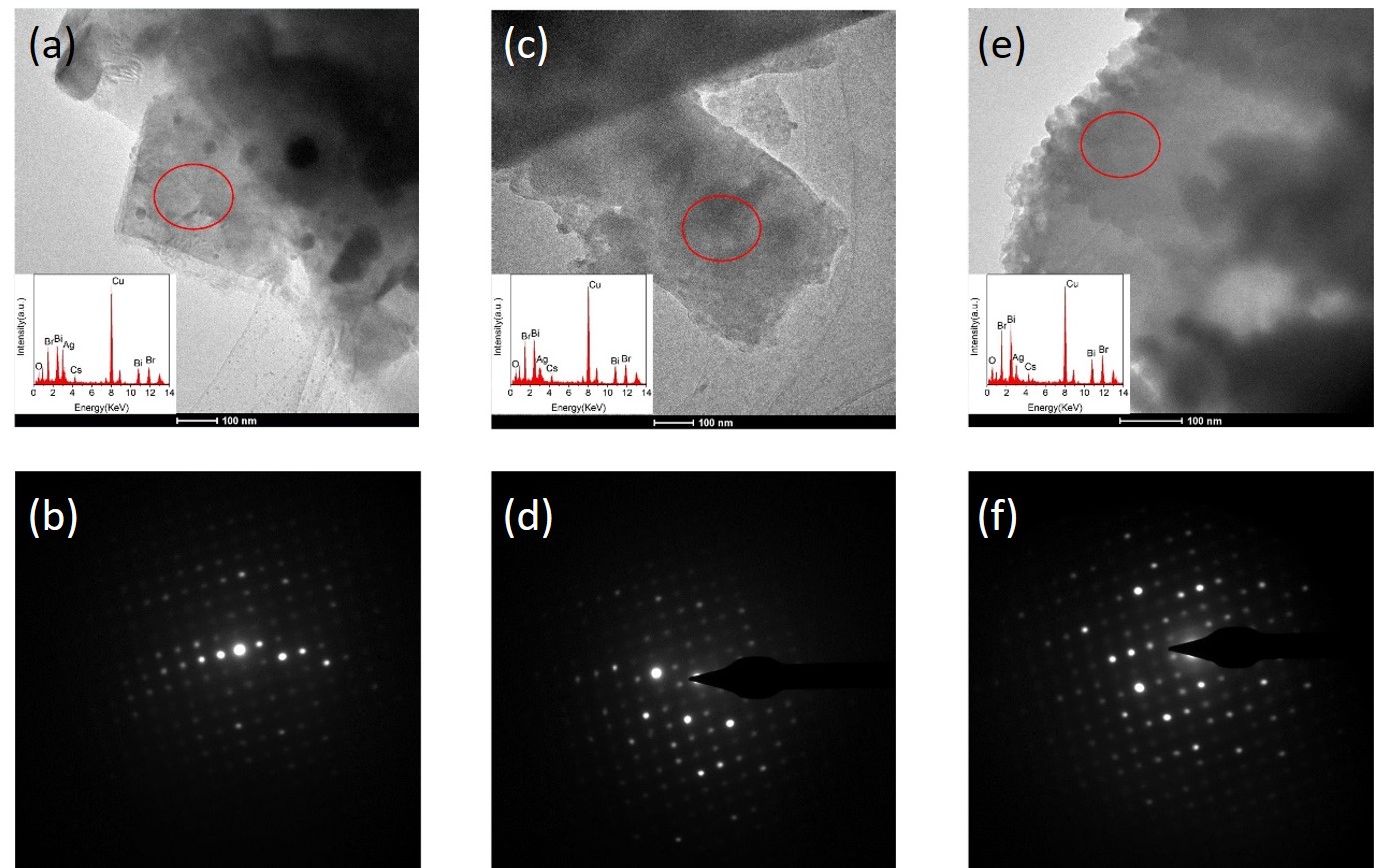
**

**Supplementary Figure 5.** TEM images and SAED patterns of the selected regions containing both BiOBr and Cs_2_AgBiBr_6_. Region 1: (a) and (b); Region 2: (c) and (d); Region 3: (e) and (f).


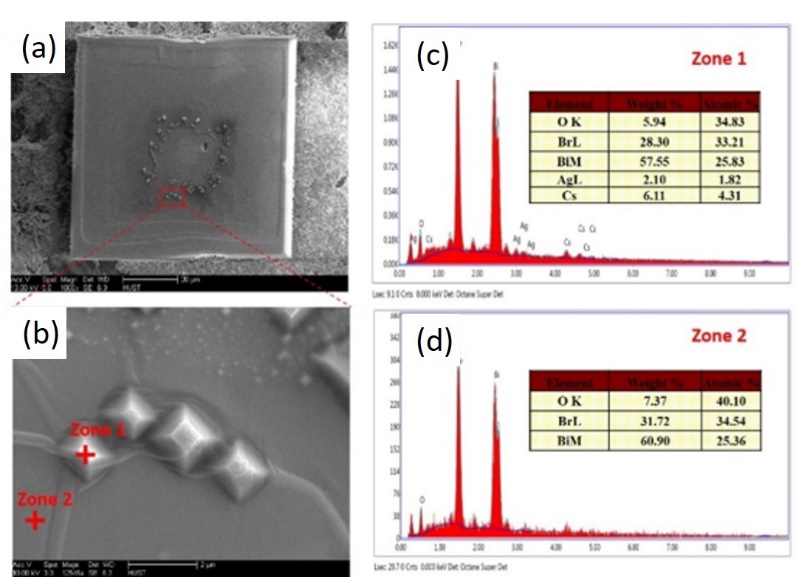


**Supplementary Figure 6** (a) SEM image of the products with BiOBr sheets as substrate for Cs_2_AgBiBr_6_ crystal growth. (b) Zoom-in image showing the detailed structures of Cs_2_AgBiBr_6_. (c) and (d) are energy dispersive spectrum for zone 1 and 2, demonstrating that zone 1 is composed of Cs_2_AgBiBr_6_ and zone 2 is BiOBr substrate. In order to verify the epitaxial relation between BiOBr and Cs_2_AgBiBr_6_, we assembled Cs_2_AgBiBr_6_ microcrystals onto BiOBr sheets. BiOBr sheets were separately prepared by hydrolysis of BiBr_3_, and then Cs_2_AgBiBr_6_ precursors in DMSO were dropped on BiOBr sheets. The rectangular sheets are BiOBr, and the top crystals are Cs_2_AgBiBr_6_. Energy dispersive spectroscopy showed different compositions for these two different zones. We could observe four truncated octahedra attached on top of the BiOBr sheets, and the alignment direction of these four octahedra is the same, demonstrating the good epitaxial growth of Cs_2_AgBiBr_6_ onto the substrate, rather than random dispersion. Additionally, the contact surface of Cs_2_AgBiBr_6_ has a rectangular shape, which is attributed to (200) facets of Cs_2_AgBiBr_6_. In contrast, (111) facets of Cs_2_AgBiBr_6_ have triangle or trapezoid shapes, which are consistent with the side surfaces of the truncated octahedra shown in the above SEM image (Supplementary Figure 6). Above growth direction is also consistent with the model shown in Fig. 2.


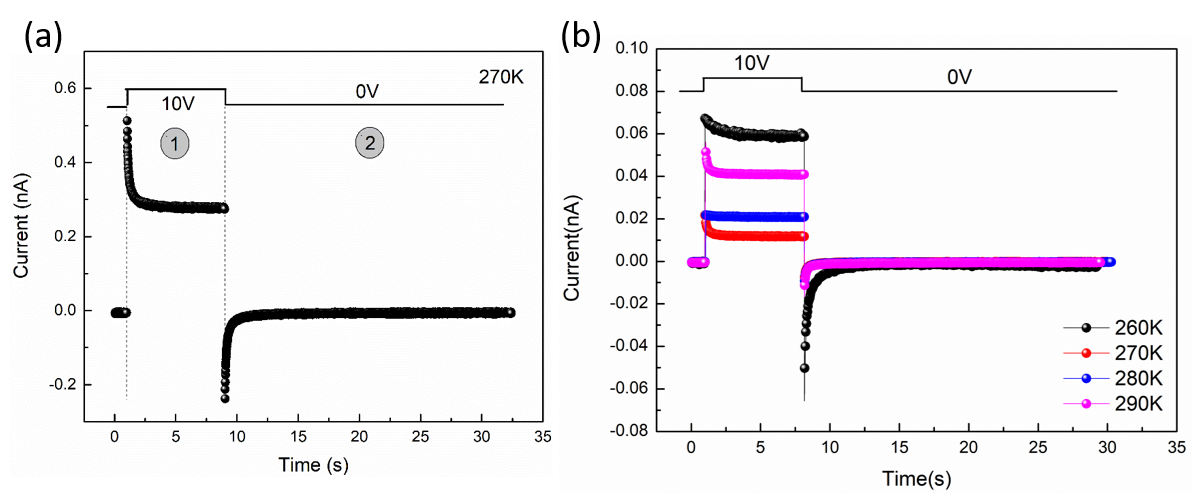


**Supplementary Figure** 7. Temporal current response curves following positive and negative biasing at 270 K. The bias sequences also display as solid lines as well. The applied external bias is 10 V. Stage 1 (**①**): the external bias is applied, and stage 2 (**②**): the external bias is removed. (b) Temporal response curves following positive and negative biasing at different temperatures from 260K to 290K.


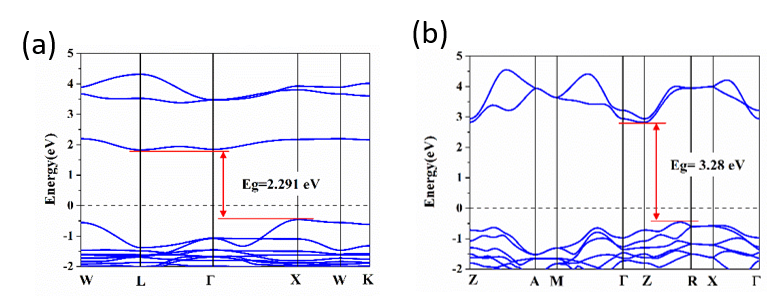


**Supplementary Figure 8.** Band structures of Cs_2_AgBiBr_6_ and BiOBr. (a) calculated by GGA-1/2 at GGA+U (U=5 eV, J=0) with SOC; (b) calculated by GGA-1/2 with SOC.

**
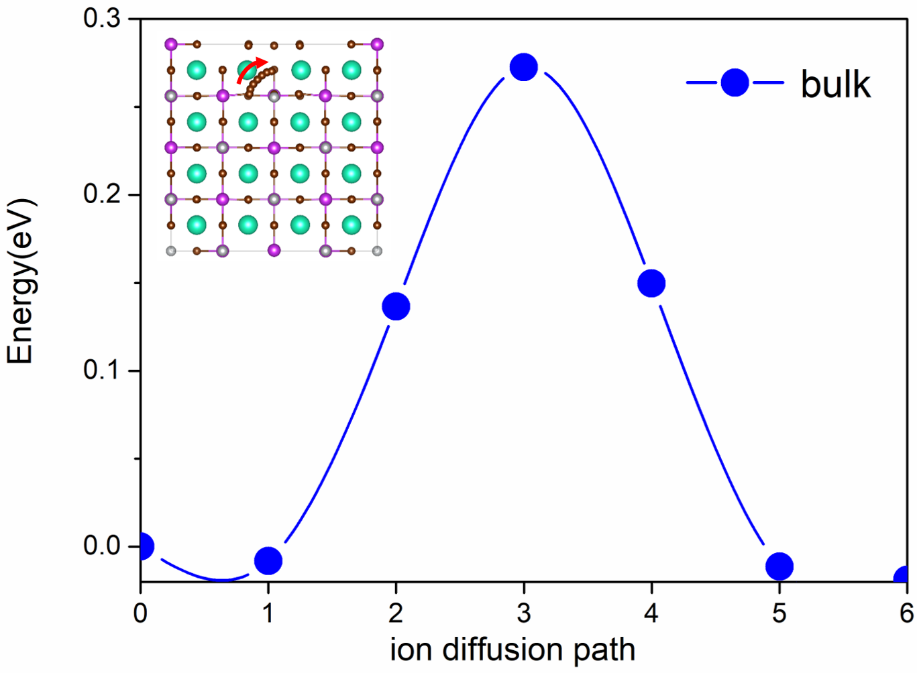
**

**Supplementary Figure 9.** Calculated energy profile along the ionic migration path for the Br^-^ vacancies in bulk Cs_2_AgBiBr_6_. The inset is migration path of Br ion vacancies, and the red arrow line represents the migration direction.


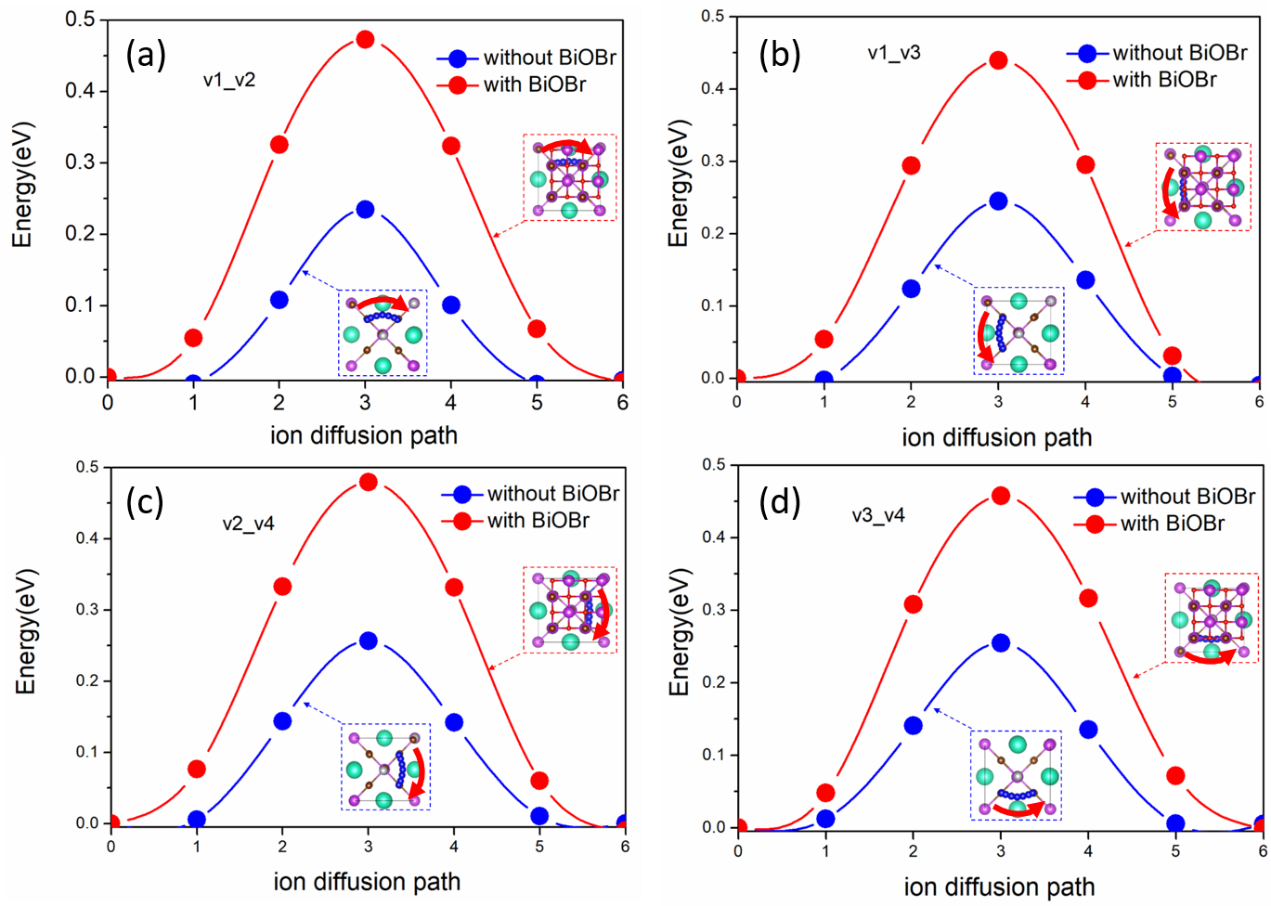


**Supplementary Figure 10.** Calculated energy profile along the ionic migration path for the Br ion vacancies in the surface of Cs_2_AgBiBr_6_ without and with BiOBr passivated. (a) migration path from v1 to v2; (b) migration path from v1 to v3; (c) migration path from v2 to v4; (d) migration path from v3 to v4. v1 to v4 are four neighbored Br ion vacancies. The insets are the ionic migration path with Br ion highlighted in blue color, and the red arrow line represents the migration direction of Br ions.


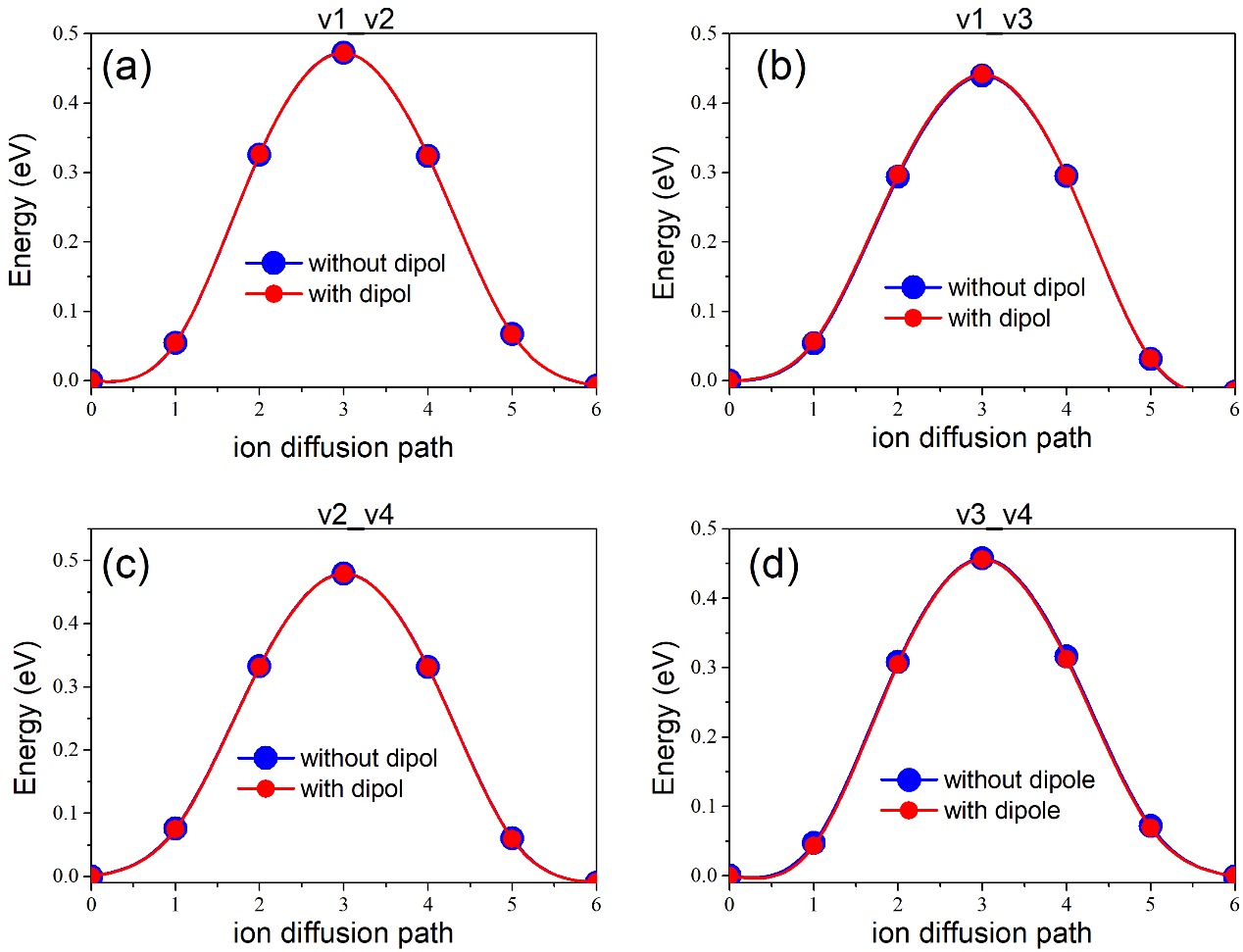


**Supplementary Figure 11.** Calculated energy profile along the ionic migration path for the Br ion vacancies on the surface of Cs_2_AgBiBr_6_ passivated by BiOBr. The blue lines/circles represent results without dipole correction, and the red lines/circles represent results considering dipole correction. (a) Migration path from v1 tov2; (b) migration path from v1 to v3; (c) migration path from v2 to v4; (d) migration path from v3 to v4. Here v1 to v4 are the four neighbored Br ion vacancies. The calculated structures are corresponding to the calculation in Supplementary Figure 10 (with BiOBr).


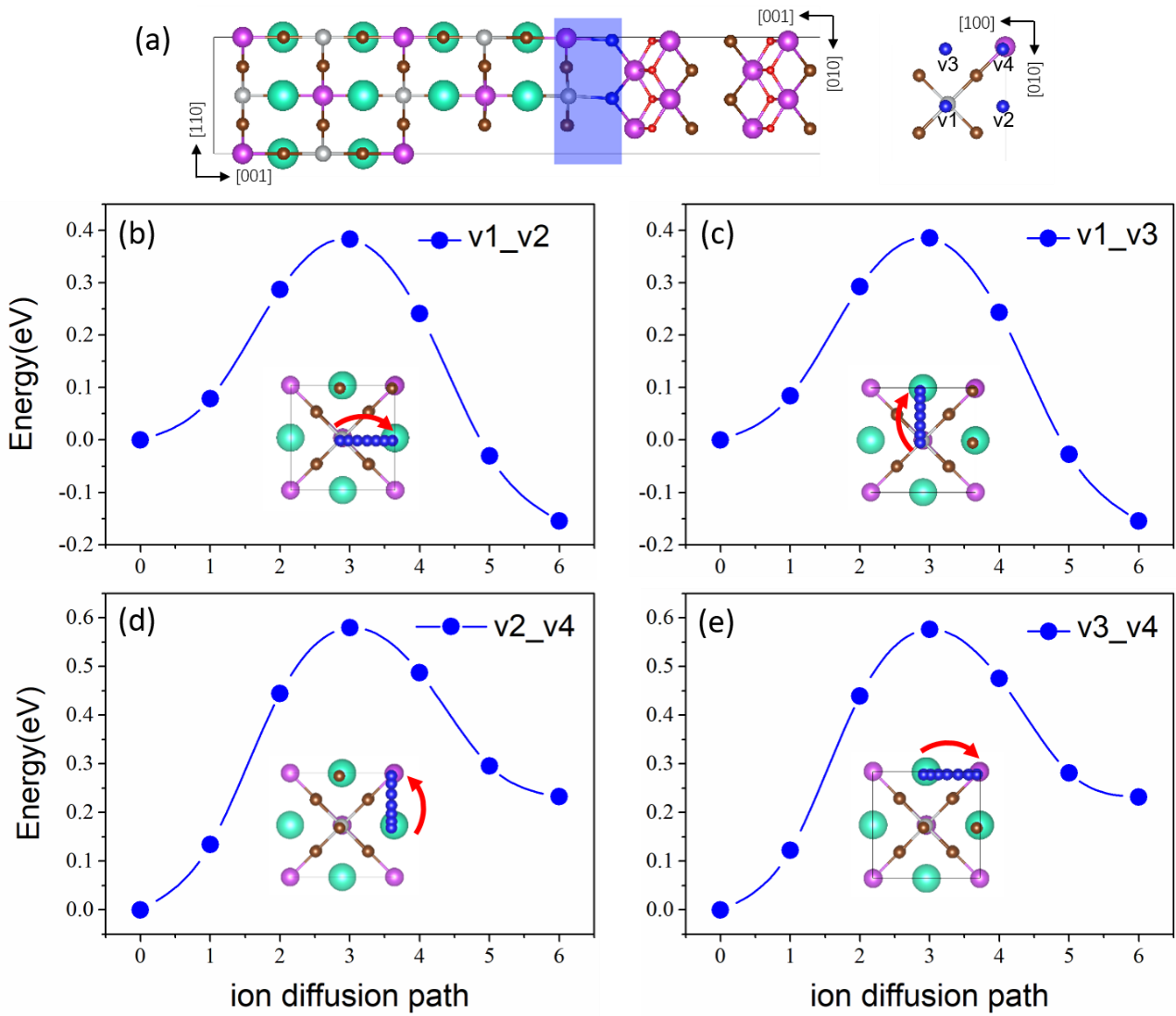


**Supplementary Figure 12.** Calculated energy profile along the ionic migration path for the Br ion vacancies at the surface of BiOBr. (a) epitaxial growth model between Cs_2_AgBiBr_6_ and BiOBr, and the blue symbols are the representative migration paths; (b) migration path from v1 to v2; (c) migration path from v1 to v3; (d) migration path from v2 to v4; (e) migration path from v3 to v4. v1 to v4 are four neighbored Br ion vacancies. The insets are the ionic migration path with Br ion highlighted in blue color, and the red arrow line represents the migration direction of Br ions.


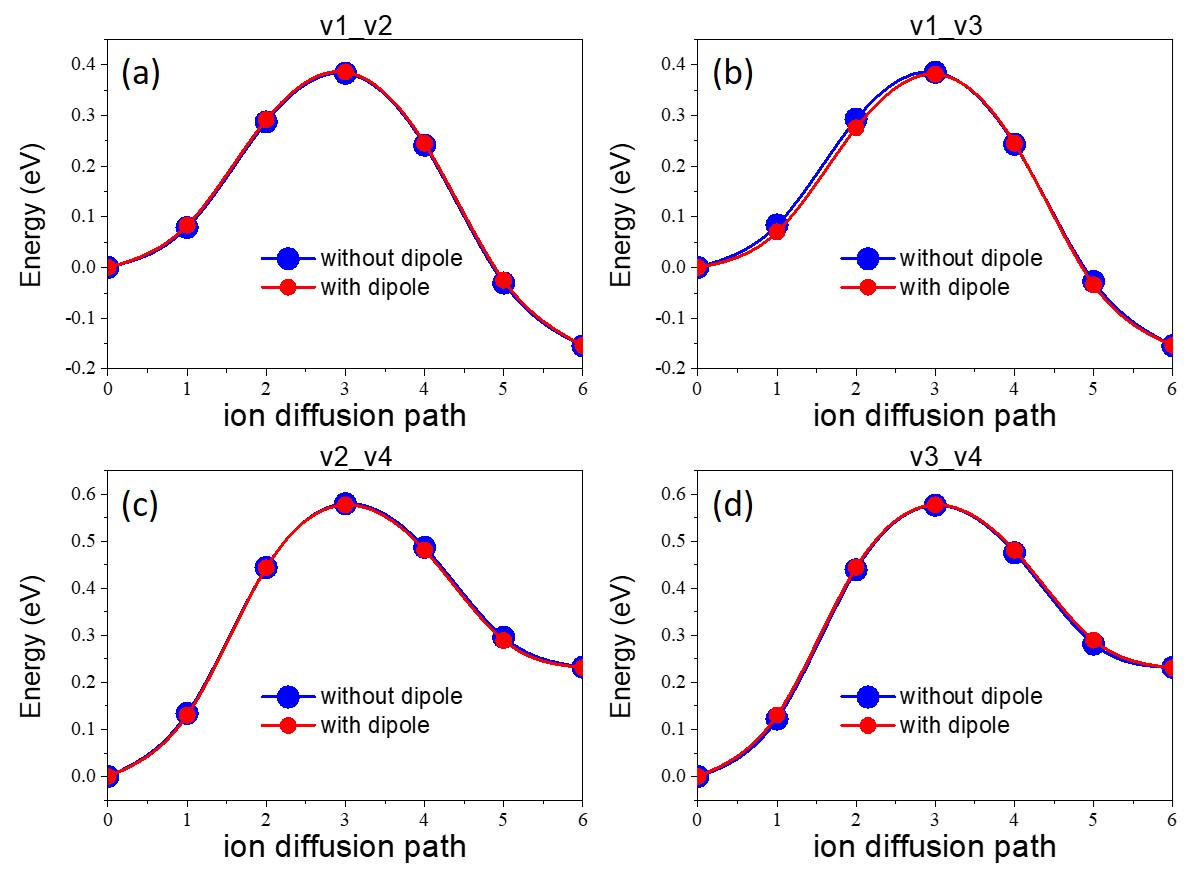


**Supplementary Figure 13.** Calculated energy profile along the ionic migration path for the Br ion vacancies at the surface of BiOBr. The blue lines/circles represent results without dipole correction, and the red lines/circles represent results considering dipole correction. (a) Migration path from v1 to v2, (b) from v1 to v3, (c) from v2 to v4, (d) from v3 to v4. Here v1 to v4 are four neighbored Br ion vacancies. (The calculated structures are corresponding to the calculation in Supplementary Figure 12)


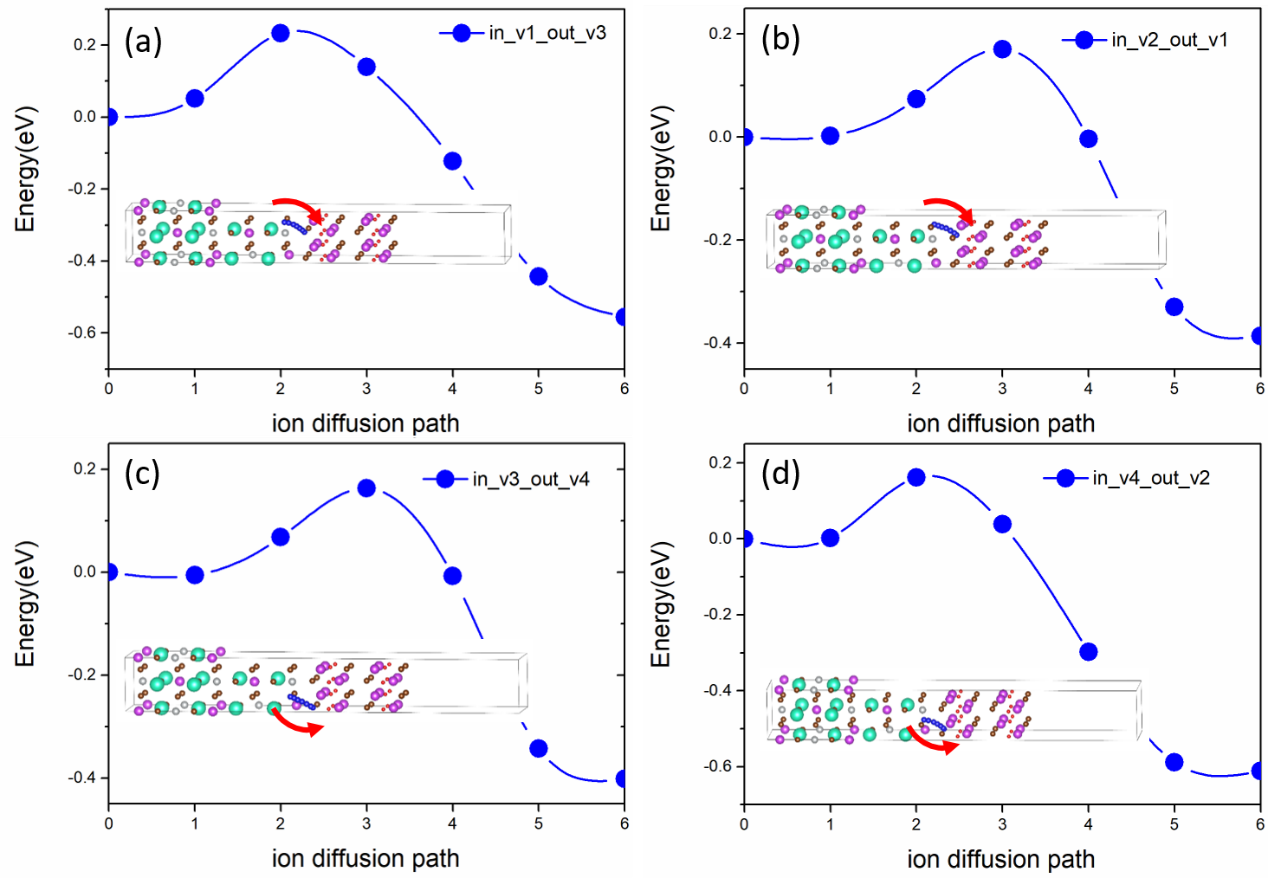


**Supplementary Figure 14.** Calculated energy profile along the ionic migration path for the Br ion vacancies across the interface between Cs_2_AgBiBr_6_ and BiOBr: (a) migration path from in_v1 to out_v3; (b) migration path from in_v2 to out_v1; (c) migration path from in_v3 to out_v4; (d) migration path from in_v4 to out_v2. Here in_v1 to v4 represent the Br ion vacancies from Cs_2_AgBiBr_6_ side, and out_v1 to v4 are the neighbored Br ion vacancies from BiOBr side. The insets are the ionic migration path with Br ion highlighted in blue color, and the red arrow line represents the ionic migration direction of Br ions.


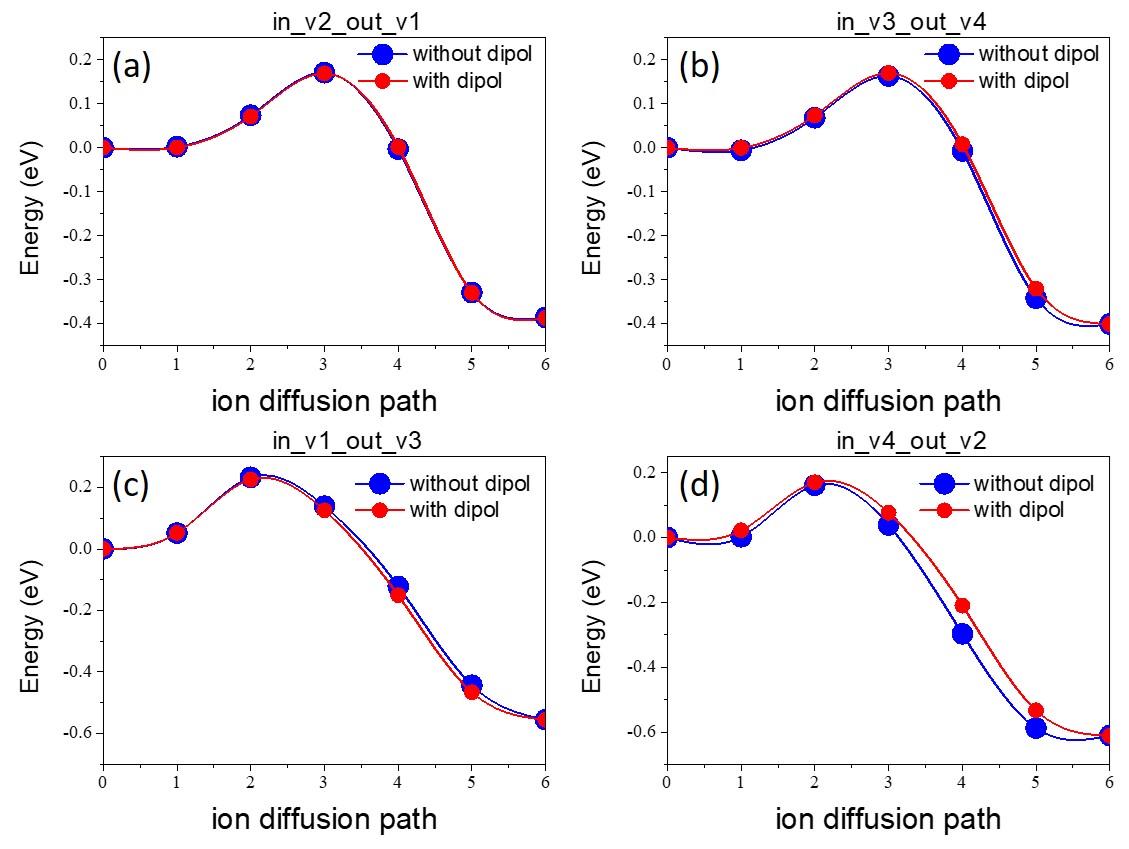


**Supplementary Figure 15.** Calculated energy profile along the ionic migration path for the Br ion vacancies across the interface between Cs_2_AgBiBr_6_ and BiOBr. The blue lines/circles represent results without dipole correction, and the red lines/circles represent results considering dipole correction. (a) Migration path from in_v2 to out_v1; (b) Migration path from in_v3 to out_v4; (c) Migration path from in_v1 to out_v3; (d) Migration path from in_v4 to out_v2. Here in_v1 to v4 represent the Br ion vacancies from Cs_2_AgBiBr_6_ side, and out_v1 to v4 are the neighbored Br ion vacancies from BiOBr side. (The calculated structures are corresponding to the calculation in Supplementary Figure 14)


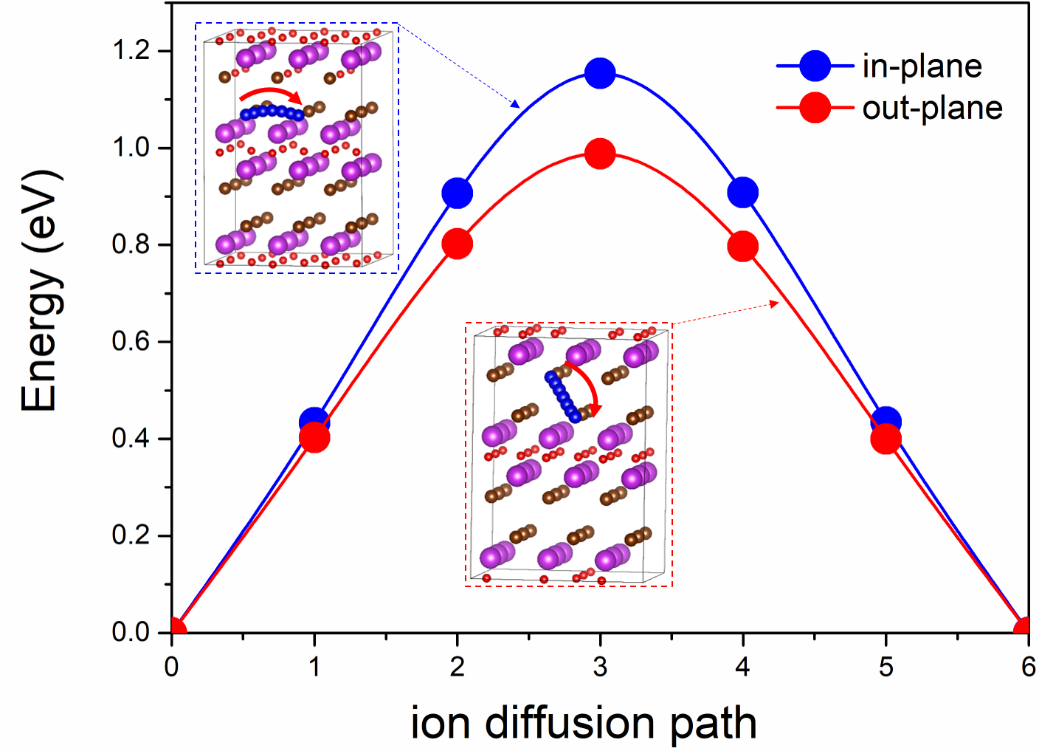


**Supplementary Figure 16.** Calculated energy profile along the ionic migration path for the Br ion vacancies in bulk BiOBr (3×3×2 supercell) and migration path of Br ion vacancies. The in-plane migration is within the BiOBr layer, and out-plane represents the intra-layer migration. In bulk BiOBr, the in-plane migration diffusion barrier of
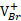
 is calculated to be 1.15 eV (blue curve), while is 0.99 eV for that of out-plane (red curve). The insets are the ionic migration path with Br ion highlighted in blue color, and the red arrow line represents the ionic migration direction of Br ions.


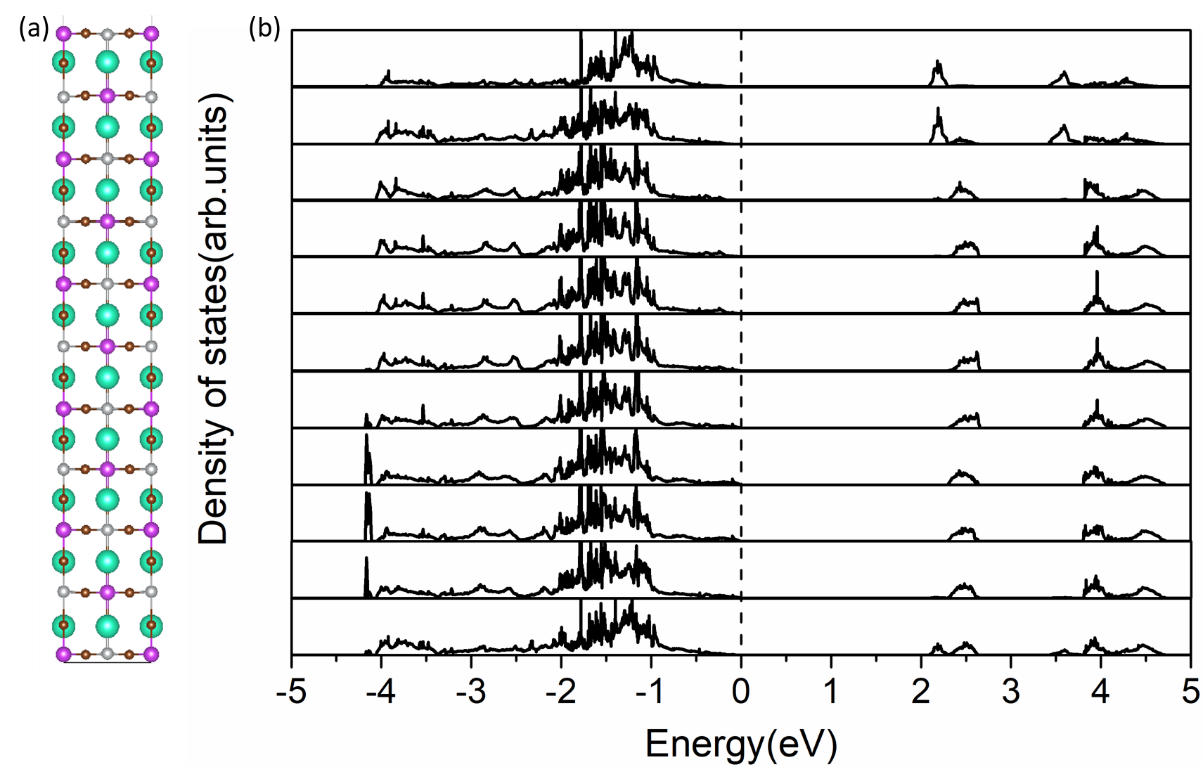


**Supplementary Figure 17.** Crystal structure of Cs_2_AgBiBr_6_ with eleven layers of (AgBr_6_)^5-^ and (BiBr_6_)^3-^ octahedra (a) and the calculated Density of states (DOS) for each layer (b).


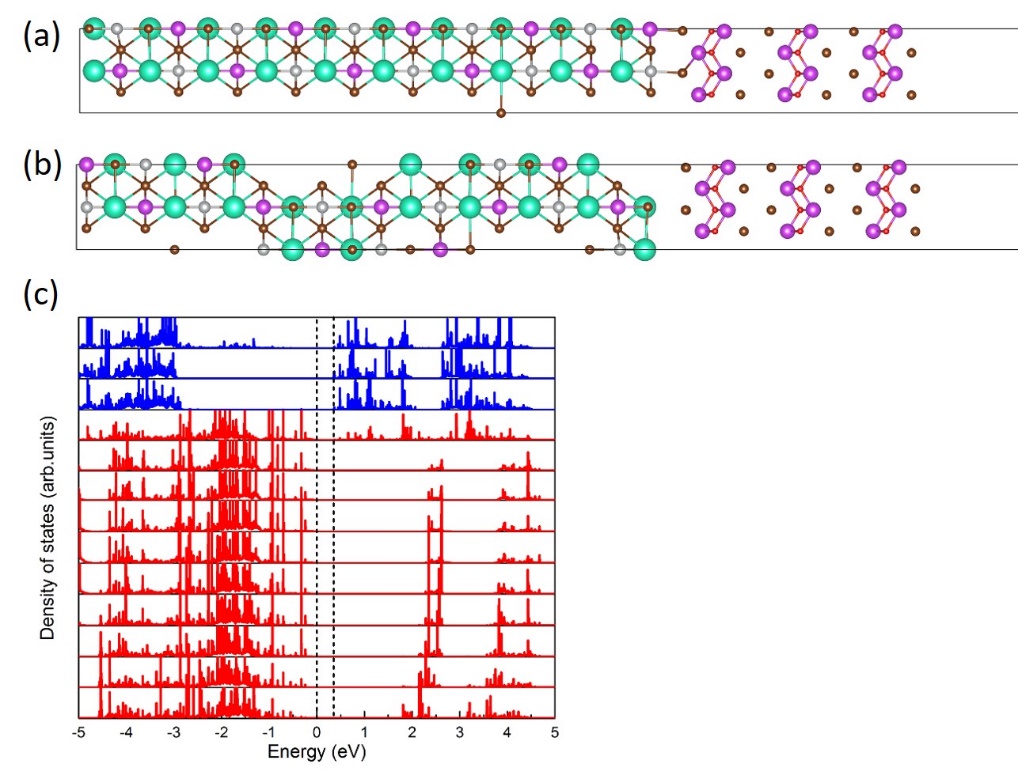


**Supplementary Figure 18.** (a) Optimized atomistic model for the Cs_2_AgBiBr_6_ (100)/BiOBr heterostructure with the type of terminations B (TB). (b) Optimized atomistic model for the Cs_2_AgBiBr_6_ (100)/BiOBr heterostructure with the type of terminations A (TA). (c) The calculated density of states (DOS) for each layer of the TA model. In Fig. 3h, we obtain the layered decomposed density of states for Cs_2_AgBiBr_6_/BiOBr heterostructures, while Cs_2_AgBiBr_6_ is terminated with CsBr (Supplementary Figure 18a). Another possible termination for Cs_2_AgBiBr_6_ is truncated BiAgBr4 octahedra (Supplementary Figure 18b). According to previous paper, the model with CsBr termination is termed as TB, and the model terminated with truncated BiAgBr_4_ octahedra is termed as TA. The Cs_2_AgBiBr_6_/BiOBr structure of TA model is also calculated, while keeping both terminations with the same number of atoms. We first compared the total energies of these two models. The results show that the energy of TB model (-660.6668 eV) is lower than that of TA model (-660.1846 eV), indicating that the TB model is more realistic. Our results are thus in line with Ref. 1. In addition, we also calculated the density of states of TA model, as shown in Supplementary Figure 18c. The results are qualitatively similar to that of TB model (Fig. 3h). Yet, the barrier between the Cs_2_AgBiBr_6_ and BiOBr layers is reduced to 0.36 eV compared with the previous 0.71 eV. Such change will not influence the main reason of this paper that BiOBr could passivate the grain boundaries of Cs_2_AgBiBr_6_ wafer and there is no additional density of states introduced within the band gaps.


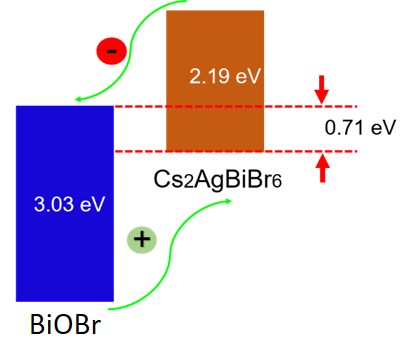


**Supplementary Figure 19.** The heterojunction band structure between Cs_2_AgBiBr_6_ and BiOBr derived from theoretical calculations. Cs_2_AgBiBr_6_ and BiOBr form a Type-II heterojunction. As there are no metallic layers, the zero energy in Fig. 3h indicates the minimum Fermi level of the system, where in reality the Fermi level typically ranges between 0 and 0.71 eV. Regardless of the fine tuning of Fermi level, a direct consequence of such electronic structure is that the excited electron is located on the BiOBr side while the hole is left on the Cs_2_AgBiBr_6_ side. Hence, the electron and the hole are spatially separated, which strongly hinders their recombination and yields a longer lifetime for the generated non-equilibrium carriers. As previously studied, the effective mass of electrons for BiOBr is around 0.3 m_0_, indicating the good electron carrier mobility within BiOBr^2^. Thereby, under bias voltage, the carrier can be transported effectively within BiOBr and Cs_2_AgBiBr_6_. The low ionic migration and good carrier mobility within BiOBr make the heterojunction structure highly resistant toward ion migration and conductive for carrier transport, which is ideal for X-ray detection applications.


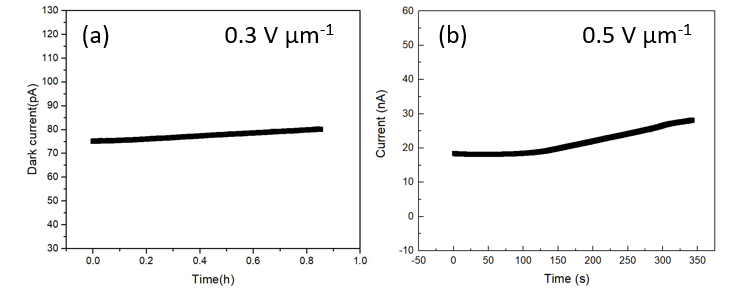


**Supplementary Figure 20.** (a) Measured dark current drift under X-ray radiation of Cs_2_AgBiBr_6_+BiOBr wafer with PI as interface layer applied an electric field of 0.3 V μm^-1^. The dark current drift is 6.7×10^-8^ nA cm^-1^ s^-1^ V^-1^. (b) The dark current drift under X-ray radiation of Cs_2_AgBiBr_6_+BiOBr wafer under an applied electric field of 0.5 V μm^-1^. The dark current drift is 7.4×10^-5^ nA cm^-1^ s^-1^ V^-1^ (as listed in Supplementary Table 2). Clearly, the dark current drift of BiOBr passivated Cs_2_AgBiBr_6_ wafer is much lower than all kinds of Pb-based perovskites.

**
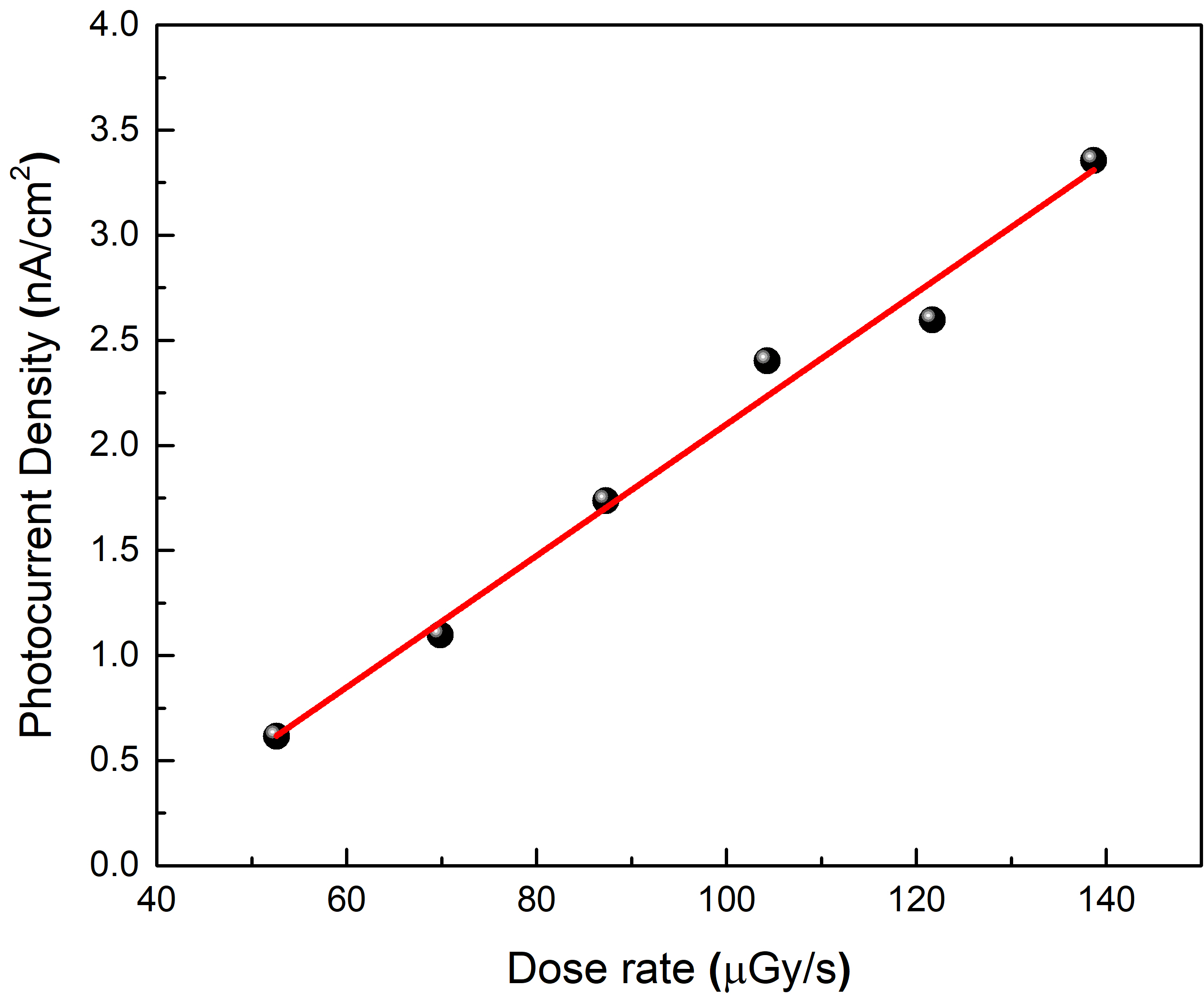
**

**Supplementary Figure 21.** The photocurrent of BiOBr-passivated Cs_2_AgBiBr_6_ wafer under different dose rates of X-ray radiations.


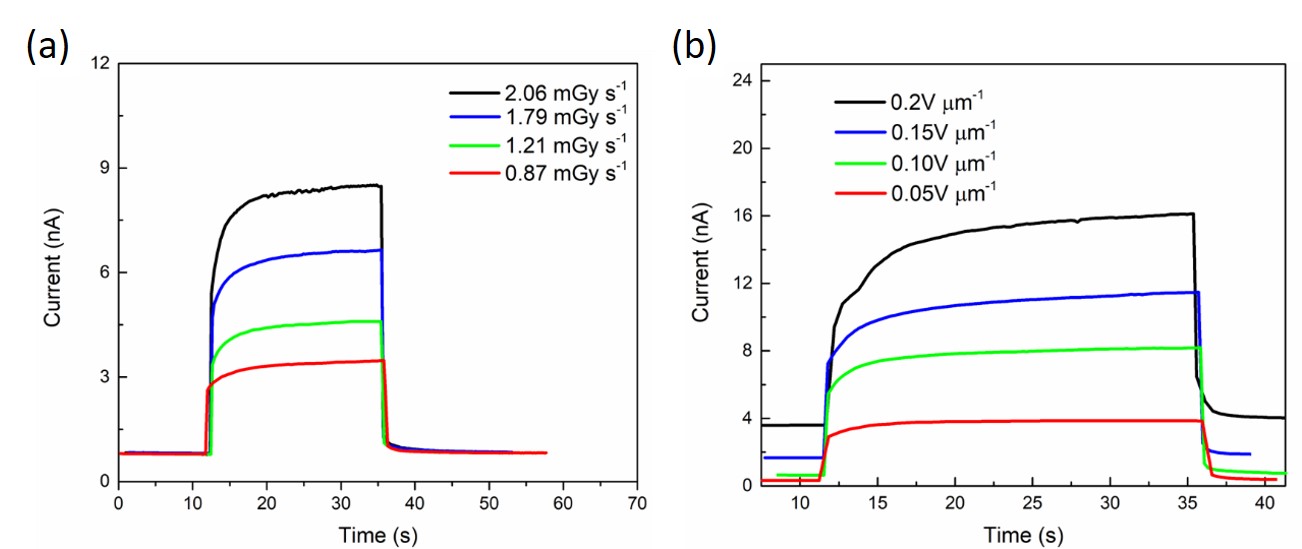


**Supplementary Figure 22.** Photocurrent response of Cs_2_AgBiBr_6_+BiOBr wafer x-ray detector at different electric field strengths and dose rates. (a) Photocurrent response toward different dose rates with a given electric field of 0.1 V μm^-1^; (b) Photocurrent response under different electric fields with a given dose rate of 2.06 mGy_air_ s^-1^.


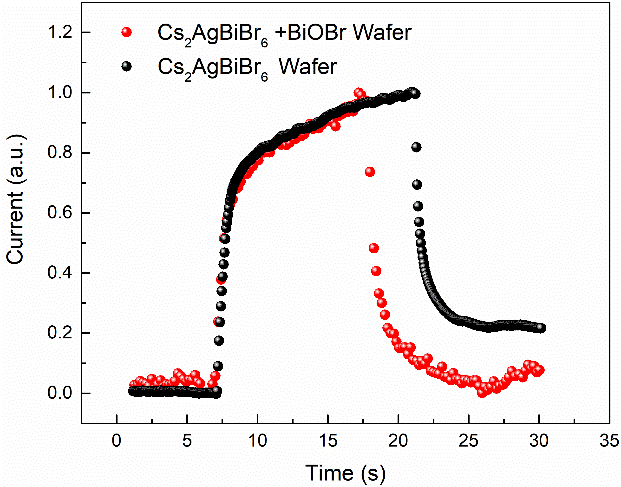


**Supplementary Figure 23.** Temporal rise behavior comparison of Cs_2_AgBiBr_6_ wafer with and without BiOBr passivation under an electric field of 0.1 V μm^-1^ and a dose rate of 138.7 μGy_air_ s^-1^.


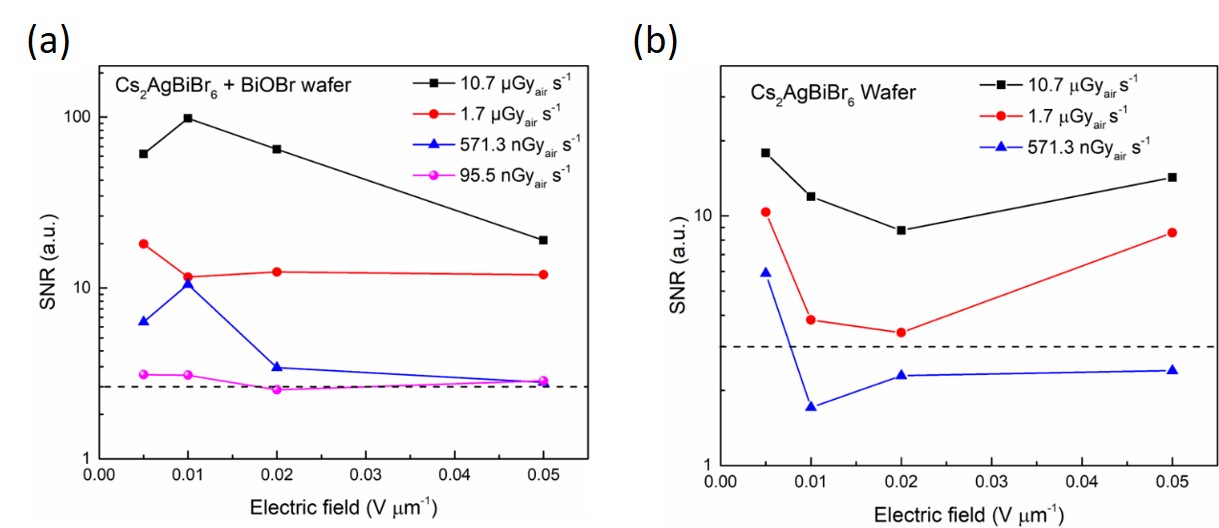


**Supplementary Figure 24.** Signal-to-noise ratio of the device. (a) Cs_2_AgBiBr_6_+BiOBr wafer; (b) Cs_2_AgBiBr_6_ wafer.


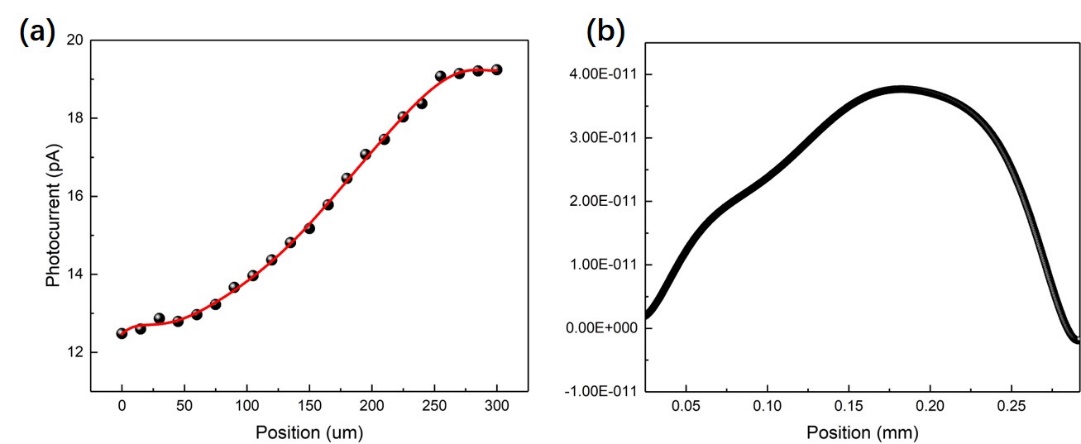


**Supplementary Figure 25.** (a) Polynomial function fit for edge spread function (
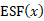
). (b) The derived line spread function (
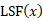
).


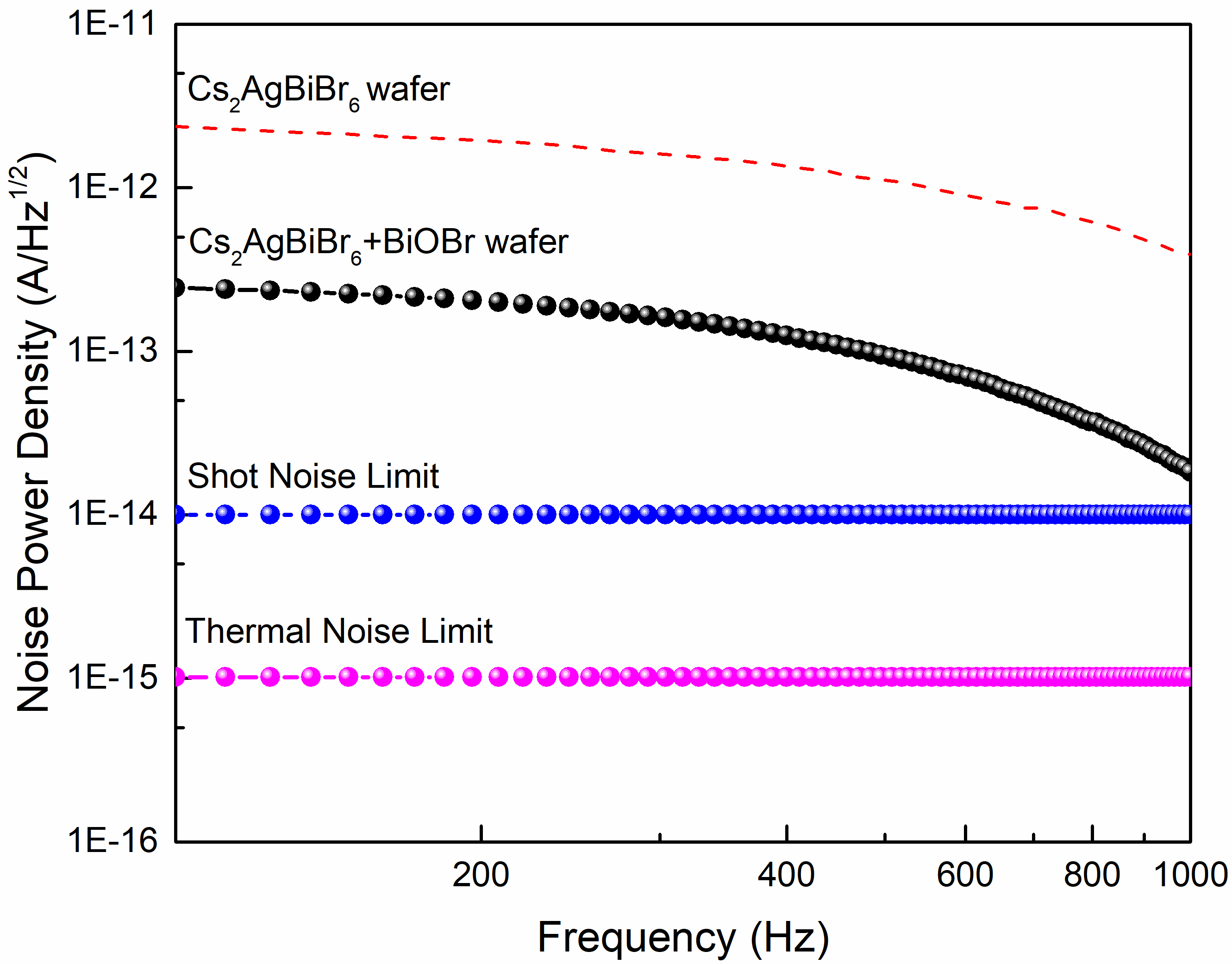


**Supplementary Figure 26.** Measured dark current noise of Cs_2_AgBiBr_6_ wafer without PI as interface layer.


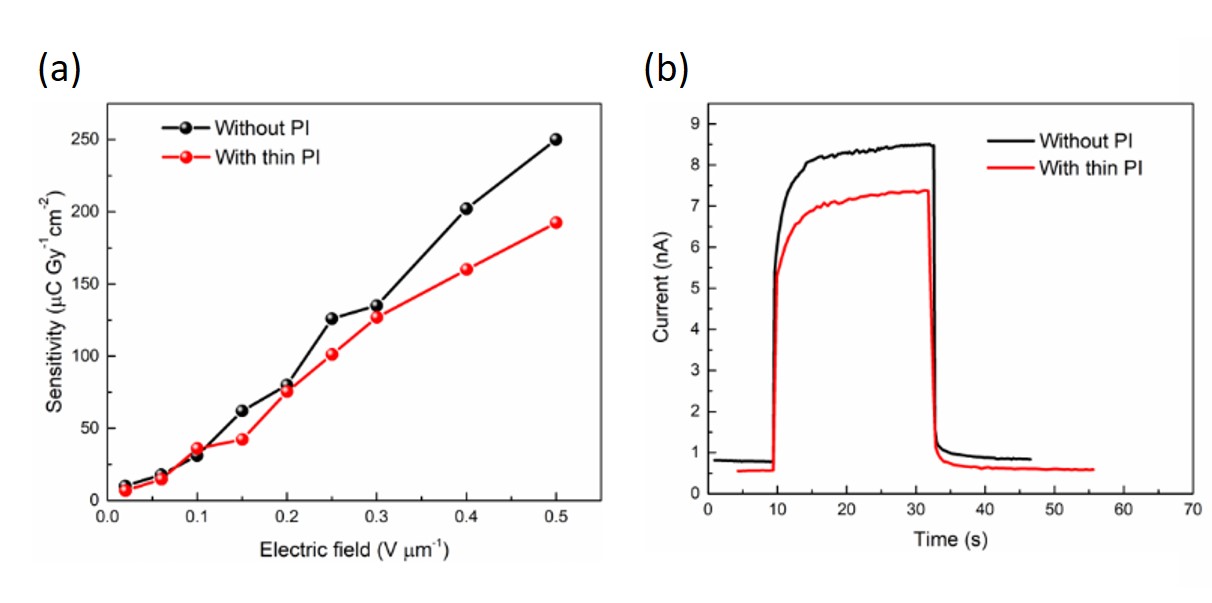


**Supplementary Figure 27.** (a) X-ray sensitivity for Cs_2_AgBiBr_6_+BiOBr wafers with/without a thin layer of PI on the surface under different electric fields. (b) Representative photocurrent response for the devices with the electric field as 0.1 V μm^-1^ and the dose rate as 2.06 mGy_air_ s^-1^.


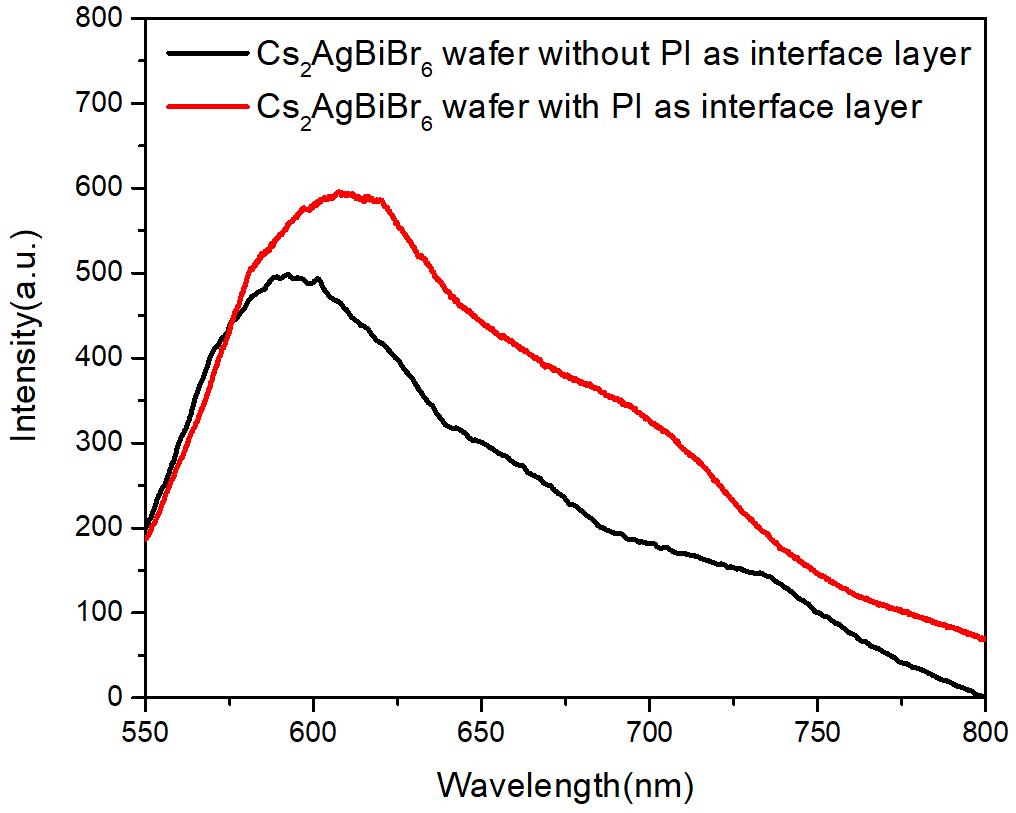


**Supplementary Figure 28.** PL (photoluminescence) spectra of Cs_2_AgBiBr_6_ wafer with/without PI as interface layer.

**Supplementary Tables**

**Supplementary Table 1.** Summary of the applied electric field and dark current drift for reported perovskite-based X-ray or γ-ray detectors.

| **Materials** | **Applied electric field**  **(V cm^-1^)** | **Dark current drift***  **(nA cm^-1^ s^-1^ V^-1^)** | **reference** |
| --- | --- | --- | --- |
| MAPbI_3_ film | 2000 | 1.7×10^-3^ | Nature Photonics, 2017, 11, 436-440. |
| CsPbBr_3_ single crystal | 2000 | 1.9×10^-4^ | Nature Communications, 2018, 9, 1609. |
| MAPbI_3_ single crystal | 100 | 1.4×10^-4^ | Nature Photonics, 2016, 10, 585-589. |
| MAPbBr_3_ single crystal | 20 | 1.2×10^-3^ | Nature Photonics, 2017, 11, 315-321. |
| BiOBr passivated Cs_2_AgBiBr_6_ film | 1000 | 8×10^-7^ | This work |
| PI coated BiOBr passivated Cs_2_AgBiBr_6_ film | 3000 | 6.7×10^-8^ | This work |
| BiOBr passivated Cs_2_AgBiBr_6_ film | 5000 | 7.4×10^-5^ | This work |

*the dark current drift is calculated according to the data in the published reports as (*I*_finish_-*I*_begin_)/(*t***s***E*), where *I*_begin_ and *I*_finish_ are the recorded initial and final dark current after a certain interval time (*t*), *s* is the pixel area, *E* is the applied electric field, respectively.

**Supplementary Notes**

**Supplementary Note 1. Experimental measurement of the activation energy of ionic migration for Cs_2_AgBiBr_6_ wafers**

The measurement strategy of ionic migrations has been documented in the previous works^3,4^. Supplementary Figure 7 displays the temporal response of current curves of the device, along with the corresponding bias sequences. After switching on the applied external bias (10 V), the dark current instantly increases and then slowly decays to a nonzero constant value. After the applied bias is removed (8s later), a current with the opposite sign is immediately observed, which gradually decays to zero over time. Such switchable behavior can be attributed to the ion migration within the Cs_2_AgBiBr_6_ wafer.

In the beginning without the external bias, V_Br_, V_Ag_, V_Cs_ and V_Bi_ are randomly distributed throughout the device channel. With the application of a positive bias, ions accumulated at the Cs_2_AgBiBr_6_ wafer/Au electrode interface. Upon turning off the external bias, ion vacancies would promptly migrate backwards. The detailed equation derivation has been studied thoroughly in our previous work^4^. The final expression is:


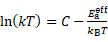
 (1)

where *C* is a fitting constant, *k* is the current decay rate, *k*_B_ is the Boltzmann constant, *T* is the temperature,
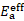
 is the effective activation energy for ion transport considering the contribution from both vacancy formation and vacancy migration. From above equation, we could derive the activation energy (
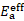
 ) by fitting the Arrhenius plots of
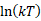
 *versus*
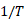
. In this work, the counter currents versus time with temperature ranging from 260 to 290 K were used for ionic migration study.

**Supplementary Note 2.** **MTF measurement and fast Fourier transformation process**

We used edge-slanted method to measure the MTF. A device with 200 μm
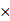
2 mm pixel was fixed on one direction scanning stage (Zolix PSA200-11-X) and scanned a line pair edge of resolution phantom. A Keithley 6517B was used to power the device and record the current. Moving the device every 15 μm and recorded X-ray response, we can plot the response of the edge. Several points were fitted by a 9 order polynomial function and got edge spread function
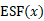
. The line spread function (LSF) can be calculated as the following equation.


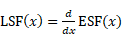
(2)

Then the
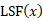
 was fitted by a Gaussian function.


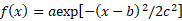
 (3)

where *a*, *b*, *c* is constant.
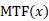
 is the Fourier transform of
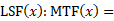
F
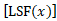


**Supplementary References**

1. Volonakis, G. & Giustino, F. Surface properties of lead-free halide double perovskites: Possible visible-light photo-catalysts for water splitting. *Appl. Phys. Lett.* **112**, 243901 (2018).
2. Ran, Z. et al. Bismuth and antimony-based oxyhalides and chalcohalides as potential optoelectronic materials. *NPJ Comput. Mater.* **4**, 14 (2018).
3. Li, D. et al. Electronic and ionic transport dynamics in organolead halide perovskites. *ACS nano* **10**, 6933-6941 (2016).
4. Pan, W. et al. Cs_2_AgBiBr_6_ single-crystal X-ray detectors with a low detection limit. *Nat. Photo.* **11**, 726 (2017).
